# Integrating univariate and multivariate stability indices for breeding clime-resilient barley cultivars

**DOI:** 10.1186/s12870-024-05530-6

**Published:** 2025-01-18

**Authors:** Ammar Elakhdar, Ahmed A. El-Naggar, Sally El-Wakeell, Anas H. Ahmed

**Affiliations:** Field Crops Research Institute, Agricultural Research Center, Giza, 12619 Egypt

**Keywords:** AMMI, Barley, GGE biplot, Multi-environment trials, Environmental variance

## Abstract

Studying genetic variability through the phenotypic performance of genotypes is crucial in the breeding program. Therefore, evaluating both yield performance and stability across diverse environments is essential in yield trials to identify high-yield potential and stable cultivars. In this study, we employed 12 univariate and 10 multivariate stability models to analyze how genotype (G), environment (E), and their interaction (G × E) affect the yield performance of 32 barley genotypes across 10 environments. The environmental main effect explained 81.3% of the total variation, compared to 18.6% for genotypes and G × E interaction effects. Using the GGE biplot ‘which-won-where’ polygon, we categorized environments into five groups and genotypes into six groups, identifying eight genotypes with mean grain yield (GY) superior to the overall mean (4.43 tons ha^− 1^). Spearman’s correlation analysis indicated significant positive correlations (*P* < 0.01) between GY and various stability parameters such as linear regression coefficients (b_i_), Perkins and Jinks’s stability parameters (B_i_), environmental variance (S_xi_^2^) and Tai’s environmental effects (α_i_), among others, as univariate stability measures. Additionally, nonparametric measures such as Nassar and Huhn’s (S^I 6^ and S^I 3^) and Thennarasu’s (NP ^I (3)^ and NP ^I (4)^), TOP-rank stability and the yield stability index (YSI), showed significant correlations with GY. Both univariate and multivariate stability models highlighted genotypes G32, G1, and G27 as the most stable, exhibiting minimal yield variation across environments. Furthermore, G15, followed by G13, G7, and G9, demonstrated high stability based on multivariate measures. Accordingly, it might be safe to utilize the stability parameters of different groups concerning static and dynamic concepts of stability to avoid the possibility of estimating the same concept of stability. This study emphasizes the importance of utilizing a combination of univariate and multivariate stability models to assess genotype stability comprehensively and select “ideal genotypes” that offer both high yield potential and stability.

**Supplementary Information:**

The online version contains supplementary material available at 10.1186/s12870-024-05530-6.

## Background

Barley (*Hordeum vulgare L*.) is among the major cereal crops and ranks fourth among wheat, rice, and maize for cultivated areas and in-field production [1]. Barley can survive environmental stressors, such as salinity and drought, and is generally cultivated on marginal lands [2, 3]. Almost 75% of the total barley production is expended for animal feed, and 25% is consumed as human food [4]. Thus, this crop is fundamental for worldwide food security. Like other cereals, environmental conditions, climate changes, and ecological conditions cause serious reductions in barley grain yield [5]. Grain yield is a complex quantitative trait that is controlled by genotype (G), environment (E), and genotype-by-environment interaction (GEI) effects [6]. The effect of GE interactions decreases the correlations between phenotypic and genotypic values; thus, the selection of superior cultivars is difficult [7]. Studying G × E interactions in multi-environment trials (METs) assists in selecting stable cultivars for different environments as well as cultivars for certain environmental conditions [8, 9].

There are two concepts of stability: dynamic and static. The static or biological concept of stability refers to Type-I stability, in which a stable genotype tends to sustain constant yield performance across a wide range of environments, with minimum environmental sensitivity; that is, genotypes show homeostasis [10]. The dynamic sense refers to the agronomical concept of stability. The analysis of dynamic concepts requires a particular set of examined genotypes, different from the measure of static stability [11]. In this context, a stable genotype has a varying response of yield performance based on the environmental conditions, which is equivalent to the mean response of the examined genotypes. This dynamic or agronomical concept of stability refers to Type II stability with high goodness of fit. Stability mainly refers to a genotype that displays a relatively constant yield regardless of varying environmental conditions, and G × E interactions are the main sources of variation in crops. According to this concept, genotypes with minimal yield variation across different environments are considered stable. In this case, stability is considered to be a static concept [12]. In some cases, breeders and agronomists do not accept this concept of stability because they prefer genotypes with high yields and are more responsive to agronomic inputs or superior environmental conditions [13]. The high-yielding performance of released cultivars is one of the most significant goals of breeders, which elucidates why they choose a dynamic idea of stability [12]. The combination of the two concepts is known as the ‘ideal genotype’ [9].

For the evaluation of yield stability, several numerical and graphical stability models, including univariate and multivariate models, have been improved for investigating the information contained in G × E interactions. Univariate or parametric methods for determining genotype stability utilize various statistical models. These include linear formulations, such as joint regression techniques incorporating parametric stability statistics like the regression slope (b_i_) [14], Eberhart and Russell’s deviation from regression (S^2^_di_) [15], and coefficient of determination (R^2^) [16]. Parallel stability approaches within regression analysis also incorporate advanced modifications like Perkins and Jinks’s stability parameters *β*_*i*_ and *D*_*ji*_ [17], Tai’s environmental effects (α_i_), and deviation from linear response (λ_i_) [18]. Additionally, methods such as Wricke’s ecovalence (W^2^_i_) [19], coefficient of variation (CV) [20], and Shukla’s stability variance (*σ*_*i*_^*2*^) [21] have been widely applied. Furthermore, environmental variance (S^2^_xi_) [22] according to Becker and Leon [12], along with genotypic stability parameters (D_i_^2^) [23], are also utilized in stability assessments. Multivariate or nonparametric models are a reasonable alternative to present parametric measurements because their expression is based on data ranks, and no assumptions are required about the distribution of homogeneity of variances and model residuals [24]. Among these models, additive main effects and multiplicative interaction (AMMI) [8] and genetics, genetics × environment (GGE) biplots [25] are commonly used for graphical evaluation of the MET dataset. These models effectively capture G × E interaction illustrating ‘which-won-where’ patterns and facilitating genotype and mega-environment analyses [8, 9]. Several specific methods within these frameworks include Kang’s rank-sum (RSM) [26], Nassar and Huhn’s nonparametric stability indices [24], Thennarasu’s nonparametric statistics [27], Ketata’s rank method [28], Fox’s TOP-rank stability parameter [29] and yield stability index (YSI) of the AMMI model [30]. These methods were employed to assess the impact of G × E interactions on yield performance [31].

Parametric analyses of phenotypic stability are mainly related to the components of variance and the distribution of genotypic, environmental, and GEI effects. These stability parameters are well characterized under confident statistical assumptions, according to the normal distribution of the errors and the effects of the GEI. However, they cannot perform correctly if those assumptions are affected by other factors, such as the existence of outliers [32]. In contrast, nonparametric measures lead to no certain modeling assumptions when relating phenotypes and environments relative to environmental factors such as abiotic and biotic stress. Therefore, each measure of univariate and multivariate models has limitations and strengths for the selection of superior cultivars and particular methods for accessing GEI effects; hence, some breeding programs integrate parameters from both parametric and nonparametric models [33].

Egypt, located in northwestern Africa near the Fertile Crescent region, faces the imperative of assessing the genetic potential of its barley collection to maximize yield development [34]. The barley breeding program in Egypt focuses on enhancing the resilience of barley cultivars against diverse environmental stresses. To achieve this goal, the program employs various breeding strategies, including the evaluation of both exotic and local materials in field trials. Exotic material nurseries, sourced annually from the International Center for Agricultural Research in the Dry Areas (ICARDA), play a pivotal role in supporting these efforts [35–37]. Each year, field nursery trials are conducted across multiple locations, including experimental farms and research stations, to assess the performance of these materials. The data obtained from these trials not only contribute to the development of high-yielding and stable barley cultivars in Egypt but also serve as valuable input for ICARDA in its collaboration to enhance the resilience of barley cultivars adapted to diverse environmental conditions.

Thus, the objectives of this study were: (a) to investigate the influence of G x E interaction on the yield performance of new advanced barley genotypes, (b) to identify high-yielding and stable genotypes across environments, and (c) to analyze the relationships among multivariate and univariate stability parameters to determine the most effective stability statistics for supporting barley breeding strategies.

## Methods

### Plant materials, experimental layout and design

**Fig. 1:**
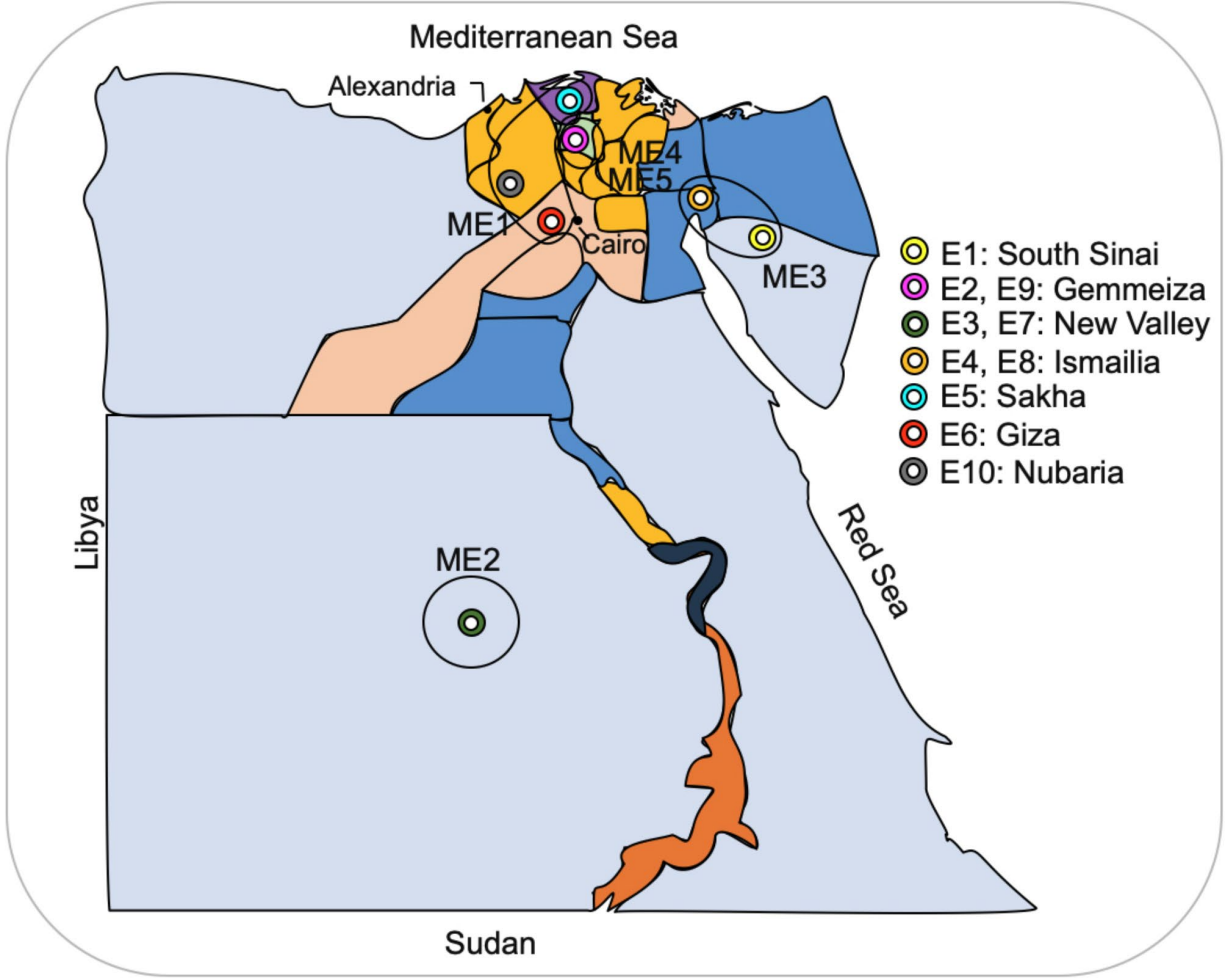
Geographical distribution of the environments studied. The ellipses show the five delineated mega-environments (MEs) based on GGE biplot information

A set of 32 six-rowed barley genotypes was evaluated as a part of field nursery trial experiments (A and B yield trials) of the Egyptian barley program. Multi-environment trial analysis (META) was carried out at seven research and farm stations of the Agricultural Research Center over two seasons (2021–2022 and 2022–2023). Each year and location were considered separate environments (Env. 1 to Env. 10) as follows: in 2021–2022, including E1; South Sinai (Ras Sidr), E2; Gemmeiza, E3; New Valley (Elkharga), and E4; Ismailia. In 2022–2023 including E5; Sakha, E6; Giza, E7; New Valley (El-Dakhla), E8; Ismailia, E9; Gemmeiza, E10; and Nubaria (Fig. 1) and (Table 1). The genotypes represented promising and advanced lines developed by the ear-to-row selection method (Supplementary file 1). The experiment was performed in a randomized complete block design (RCBD) with three replications. Each plot had four rows that were 2 m long with a spacing of 30 cm. Agronomic practices were treated as non-experimental variables and applied uniformly to the entire experimental area. The data were recorded in the middle rows to reduce the experimental error (the grain yield measured from a net plot size of 2 m^2^ was converted into tons ha^− 1^).

**Table 1:** Characterization and description of test environments

							Temperature (C°)			
Env.	Location	Years	Longitude	Latitude	Precipitation (mm)	Irrigation	Mix.	Max.	mean	Soil type
E1	South Sinai	2022	32.155	29.333	0.512	-	9.68	23.28	15.76	Sandy
E2	Gemmeiza	2022	30.955	31.133	0.638	applied	12.48	26.78	18.38	Clay
E3	New Valley	2022	30.506	25.266	0.0011	applied	12.36	27.70	19.48	Sandy loam
E4	Ismailia	2022	32.44	30.66	0.397	-	11.99	25.10	17.73	Sandy loam
E5	Sakha	2023	30.565	31.529	1.200	applied	16.12	21.62	18.56	Clay
E6	Giza	2023	31.51	30.10	0.256	applied	12.15	26.05	17.68	Clay
E7	New Valley	2023	30.506	25.266	0.0032	applied	11.35	26.34	18.30	Sandy loam
E8	Ismailia	2023	32.44	30.66	0.397	-	11.99	25.10	17.73	Sandy loam
E9	Gemmeiza	2023	30.955	31.133	0.758	applied	11.50	24.60	17.20	Clay
E10	Nubaria	2023	31.22	30.05	0.543	applied	10.49	24.24	16.70	Sandy loam

Env.; Environments, Min. minimum, Max. maximum. Ave. Average. Maximum and minimum temperatures during the growing season (°C)

### Statistical analysis

#### Analysis of variance

The analysis of variance (ANOVA) for grain yield was performed using the general linear model (GLM) method, where the total variation was divided into components of G, E and G × E interaction effects. The environmental effect was treated as random, and the genotype was treated as a fixed effect. The effect of environment was verified against the replications of the environment as error 1, the effect of genotype was verified against the G × E interaction, and the G × E interaction was verified against pooled error as error 2. The ANOVA model for the genotype yield value $\:{\text{Y}}_{\text{i}\text{j}\text{k}}$ of the *i*_th_ level of the genotype (G) and *j*_th_ level of the location (L), excluding the blocking factor, is:

$\:{\text{Y}}_{\text{i}\text{j}\text{k}}=m+{G}_{\text{i}}+{L}_{j}+{\left(GL\right)}_{\text{i}\text{j}}+{e}_{ijk}$, where $\:m$ is the grand mean and $\:{e}_{ijk}$ is the random error.

#### Estimation of yield stability parameters

The GGE biplot was estimated according to the symmetrical scaling method, which uses singular value partitioning of the first two principal components analysis (IPCA) [38]. Using the GGE biplot method, several graphical analyses comprising genotype evaluation, mega-environment analysis and discriminative power of exam environments against their representativeness ability were investigated. The yield stability index (YSI) of the AMMI model [30] was used to partition the grain yield variation into the AMMI stability value (ASV) and AMMI effects [31]. Gollob’s F test [39] was used to preserve the number of significant interactions with IPCA in the model. AMMI and GE biplot analyses were performed by using GenStat software [40]. To assess genotype stability, we employed 12 univariate and 10 multivariate models to analyze the impact of genotype (G), environment (E), and their interaction (G × E) on the grain yield of 32 barley genotypes (Table 2 and Supplementary file 2). The univariate models included measures such as mean grain yield (GY; tons ha^− 1^), coefficient of variability (CV) [20], linear regression coefficients (b_i_) [14], Eberhart and Russell’s deviations from regression (S^2^_di_) [15], coefficient of determination (R^2^) [16], Shukla’s stability variance (σ_**i**_^**2**^) [21], Perkins and Jinks’s stability parameters (*β*_*i*_ and *Dji*) [17], Wricke ecovalence (W^2^_i_) [19], Roemer’s environmental variance (S_xi_^2^) [22], Tai’s environmental effects (α_i_) and deviation from the linear response (λ_i_) [18], Hanson genotypic stability (D_i_^2^) [23]. Additionally, multivariate stability models included AMMI stability value (ASV) [31], Kang’s rank-sum (RSM) [26], Nassar and Huhn’s (S^I 3^ and S^I 6^) [24] and Thennarasu’s (NP^I (3)^ and NP^I (4)^) [27] nonparametric statistics, yield stability index (YSI) [30], Ketata’s plotting mean rank (kr) [28], and Fox’s TOP-rank stability (TOP) [29].

**Table 2:** List of parametric and nonparametric stability statistics used to analyze the effects of GEIs in MET experiments

Stability parameter	Symbol	Stability concept	Pattern of selection	References
Coefficient of variability	CV	Biological	small values	[20]
Shukla’s stability variance	σ_i_^2^	Agronomic	small values	[21]
Join regression	*βi*	Biological- Agronomic	small values	[17]
	*Dji*	Agronomic	values close to 1	
Deviation mean squares	b_i_	Biological- Agronomic	values close to 1	[15]
	S^2^_di_	Agronomic	small values	
Coefficient of regression	*b*_*i*_		small values	[14]
Coefficient of determination	*R* ^*2*^	Agronomic	small values	[16]
Ecovalence	W^2^_i_	Agronomic	small values	[19]
Environmental variance	S_xi_^2^		small values	[22]
Tai’s stability statistics	λ and α		small values	[18]
Genotypic stability	D_i_^2^		small values	[23]
AMMI stability value	ASV		small values	[31]
Yield stability index	YSI		Maximum value	[30]
Nassar and Huhn’s variance of rank	S^I (3, 6)^		small values	[24]
Thennarasu’s nonparametric statistics	NP^I (3, 4)^		small values	[27]
Fox’s TOP-rank				[29]
Kang’s rank-sum	RSM		small values	[26]
Ketata’s Plotting mean rank	kr			[28]
	σ_gy_			

#### Spearman’s rank correlation and clustering

Spearman’s rank correlation was calculated on the ranks to measure the relationships between the parameters. To better understand the relationships among the models, a hierarchical cluster analysis based on non-weighted values of 32 barley genotypes was performed. The squared Euclidean distance was used as a dissimilarity measure required in Ward’s method [41].

## Results

### AMMI analysis

#### Analysis of variance for the yield

**Fig. 2:**
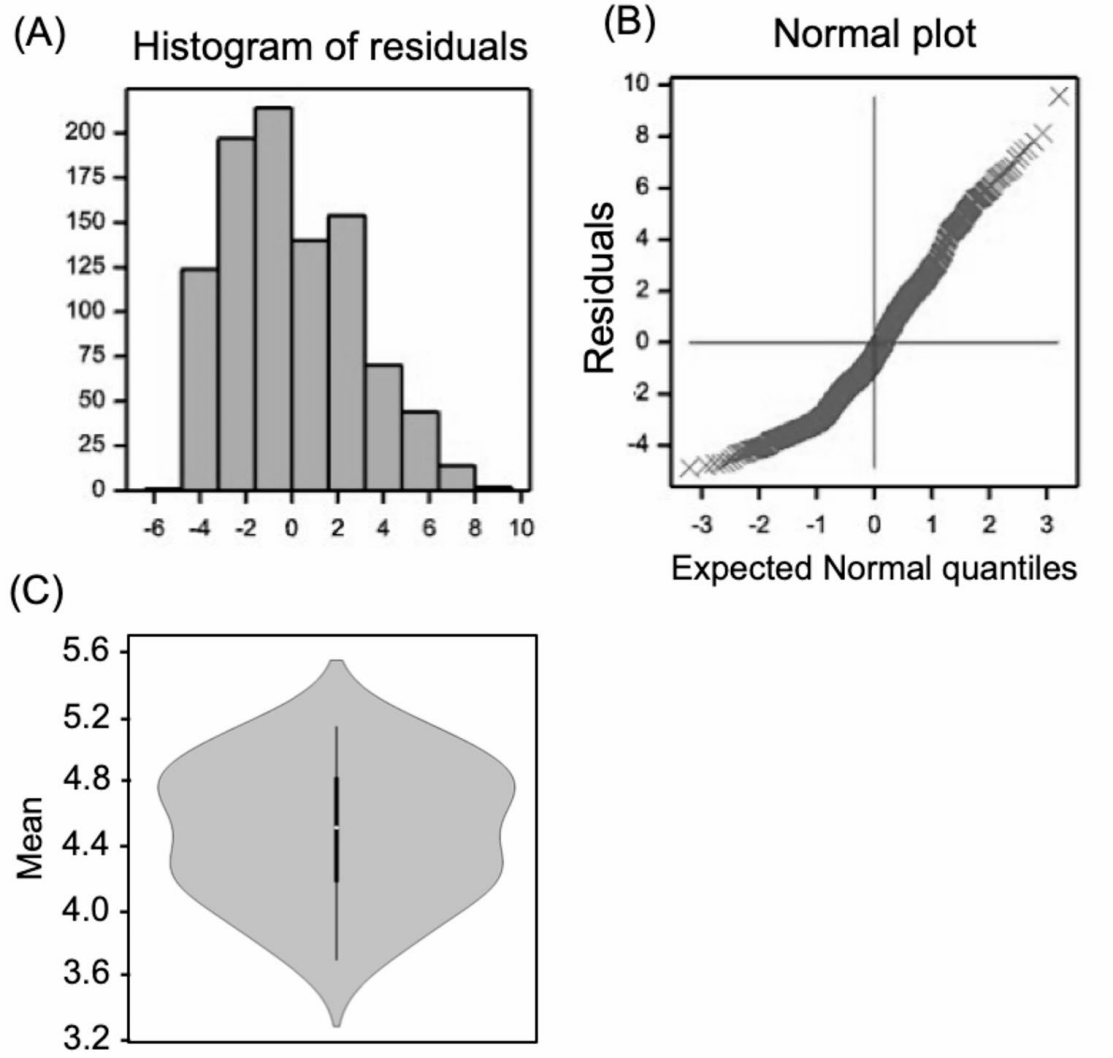
A panel of diagnostic plots for grain yield. (**A**) Histogram of residuals indicating a normal distribution of the residuals. (**B**) The normal probability plot displays the distribution of the residual values and their linear pattern. (**C**) Violin plot showing the distribution of grain yield across environments

Simple residual diagnostic plots revealed that grain yield values were normally distributed and had constant variance (Fig. 2). The ANOVA results revealed that environmental effects accounted for 81.3% of the total variance, while genotype and GEI contributed 2.9% and 15.7%, respectively (Table 3, Supplementary file 3). The GEI effect was approximately five times greater than the genotype effect, significantly impacting mean yield performance. This is supported by GEI effects on mean yield, ranging from 0.21 tons ha⁻¹ for genotype G16 in E1 to 10.13 tons ha⁻¹ for G15 in E5, while genotype effects varied from 3.66 tons ha⁻¹ for G24 to 5.10 tons ha⁻¹ for G18 (Table 4). A high level of variability in mean grain yield was detected across environments, ranging from 0.96 tons ha⁻¹ in E1 to 9.37 tons ha⁻¹ in E5. The low yield in E1 was due to severe drought stress, caused by low precipitation and lack of supplemental irrigation (Table 1). This significant variation indicates that the environments studied represent distinct agroclimatic conditions. The effects of GEI on mean grain yield were also evident in the box plot (Fig. 2).

**Table 3:** Analysis of additive main effects and multiplicative interaction effects on the grain yield of 32 barley genotypes grown in 10 test environments

SOV	df	SS	MS	F Cal	Explained (%)
Environments (E)	9	3648.8	405.42	822.58	81.3
Genotypes (G)	31	131.9	4.25	8.63	2.9
G x E	279	706.4	2.53	5.14	15.7
IPCA 1	39	252.3	6.47	13.68	35.7
IPCA 2	37	131.9	3.57	7.54	18.7
IPCA 3	35	107.7	3.08	6.51	15.2
IPCA 4	33	72	2.18	4.61	10.2
IPCA 5	31	57.9	1.87	3.95	8.2
IPCA 6	29	46.7	1.61	3.41	6.6
IPCA 7	27	22.6	0.84	1.77	3.2
IPCA 8	25	10.7	0.43	0.91	1.5
IPCA 9	23	4.5	0.2	0.41	0.6
Residuals	640	315.43	0.49	NA	0

**Table 4:** Mean grain yield (tons ha^−^1) values of genotype, environment, and GE interaction effects for 32 barley genotypes grown in 10 test environments

Genotype	E1	E2	E3	E4	E5	E6	E7	E8	E9	E10	Mean (tons ha^− 1^)
G1	0.63	6.83	4.20	1.08	8.00	6.20	2.70	2.77	2.67	4.52	3.96
G2	0.54	7.00	6.50	1.13	9.00	4.77	4.94	2.97	3.37	5.91	4.61
G3	0.55	10.00	3.25	1.35	8.38	6.08	1.35	2.75	2.82	5.48	4.20
G4	0.58	7.00	5.33	1.43	7.13	5.96	2.30	3.63	3.74	4.21	4.13
G5	1.35	5.25	4.83	1.34	6.50	5.15	1.67	3.16	3.02	5.83	3.81
G6	0.88	3.83	3.38	1.36	9.38	5.01	5.40	2.95	2.77	6.67	4.16
G7	1.40	5.83	4.37	1.26	8.90	7.63	3.90	3.03	4.19	6.59	4.71
G8	0.50	5.67	6.00	1.35	8.58	6.67	2.54	2.25	2.28	5.67	4.15
G9	1.40	6.83	5.58	1.38	9.25	7.08	4.29	3.04	2.82	5.67	4.73
G10	0.65	5.42	7.25	1.68	9.88	6.08	3.67	3.86	3.49	6.51	4.85
G11	1.23	6.67	3.13	1.41	8.88	5.72	2.86	3.02	2.11	6.91	4.19
G12	1.23	6.42	4.72	1.43	9.70	6.91	5.00	2.07	1.97	6.63	4.60
G13	0.98	9.08	6.00	1.15	8.80	7.37	3.97	1.57	3.13	6.27	4.83
G14	1.50	9.58	3.33	1.03	9.99	6.34	2.78	2.80	5.87	7.35	5.06
G15	1.30	8.00	3.83	1.15	10.13	7.94	3.10	2.91	3.38	6.14	4.78
G16	0.21	10.17	3.37	1.08	8.00	5.67	5.17	2.55	4.21	7.02	4.74
G17	1.30	7.30	3.98	1.50	8.28	5.06	5.71	3.14	4.21	4.74	4.52
G18	0.43	9.17	5.21	1.05	10.08	7.08	4.56	2.12	4.21	7.08	5.10
G19	0.90	10.03	3.35	1.28	8.38	6.82	1.90	3.00	4.69	4.94	4.55
G20	1.33	5.80	2.92	1.17	9.53	6.53	2.94	2.50	3.83	6.10	4.26
G21	0.63	8.08	6.00	1.39	10.38	7.39	3.10	1.91	3.92	6.15	4.89
G22	0.88	7.83	4.13	1.69	9.13	6.27	4.44	1.93	3.53	4.79	4.46
G23	0.48	7.75	5.00	1.00	9.75	4.29	2.14	2.41	2.33	5.98	4.11
G24	0.60	7.83	2.90	1.43	7.33	4.53	1.83	2.70	2.67	4.76	3.66
G25	0.78	9.58	3.50	1.33	8.75	2.37	3.41	1.53	2.33	4.25	3.78
G26	1.38	6.83	5.25	1.55	9.75	3.10	2.70	2.10	2.16	4.21	3.90
G27	0.98	6.58	3.25	1.22	9.08	5.84	3.25	2.56	3.21	5.68	4.16
G28	1.30	9.00	6.25	1.13	9.88	6.39	2.14	1.98	3.13	6.67	4.79
G29	1.03	8.33	4.20	1.30	9.38	5.15	2.62	2.36	2.91	7.46	4.47
G30	1.05	8.75	3.75	1.01	8.50	6.72	2.86	1.51	3.35	5.95	4.34
G31	1.33	7.25	3.75	1.15	9.88	6.27	2.78	1.83	4.17	6.90	4.53
G32	1.30	7.50	5.38	1.20	9.33	6.41	3.25	3.48	3.89	5.79	4.75
Total	30.65	241.49	143.90	40.98	299.83	190.80	105.25	82.39	106.38	188.85	
Mean	0.96	**7.54**	4.50	1.28	**9.06**	**5.96**	3.29	2.57	3.32	5.90	4.43
Env. Index	-3.52	3.13	0.02	-3.20	4.89	1.49	-1.19	-1.90	-1.15	1.42	

E1: South Sinai (2021–2022), E2: Gemmeiza (2021–2022), E3: New Valley (2021–2022), E4: Ismailia (2021–2022), E5: Sakha (2022–2023), E6: Giza (2022–2023), E7: New Valley (2022–2023), E8: Ismailia (2022–2023), E9: Gemmeiza (2022–2023), E10: Nubaria (2022–2023)

The GEI comprises nine IPCA components, with weights represented by the sum of squares (SS) and decreasing significance. F-tests estimated the significance of these components at the 0.01 probability level (Table 3). Splitting GEIs into multiplicative components showed that the first four IPCAs were significant (*p* < 0.01), increasing GE interaction variance by 69.6%. The first three IPCAs accounted for 35.7%, 18.7%, and 15.2% of the total variation, respectively (Table 3).

#### AMMI Biplot

**Fig. 3:**
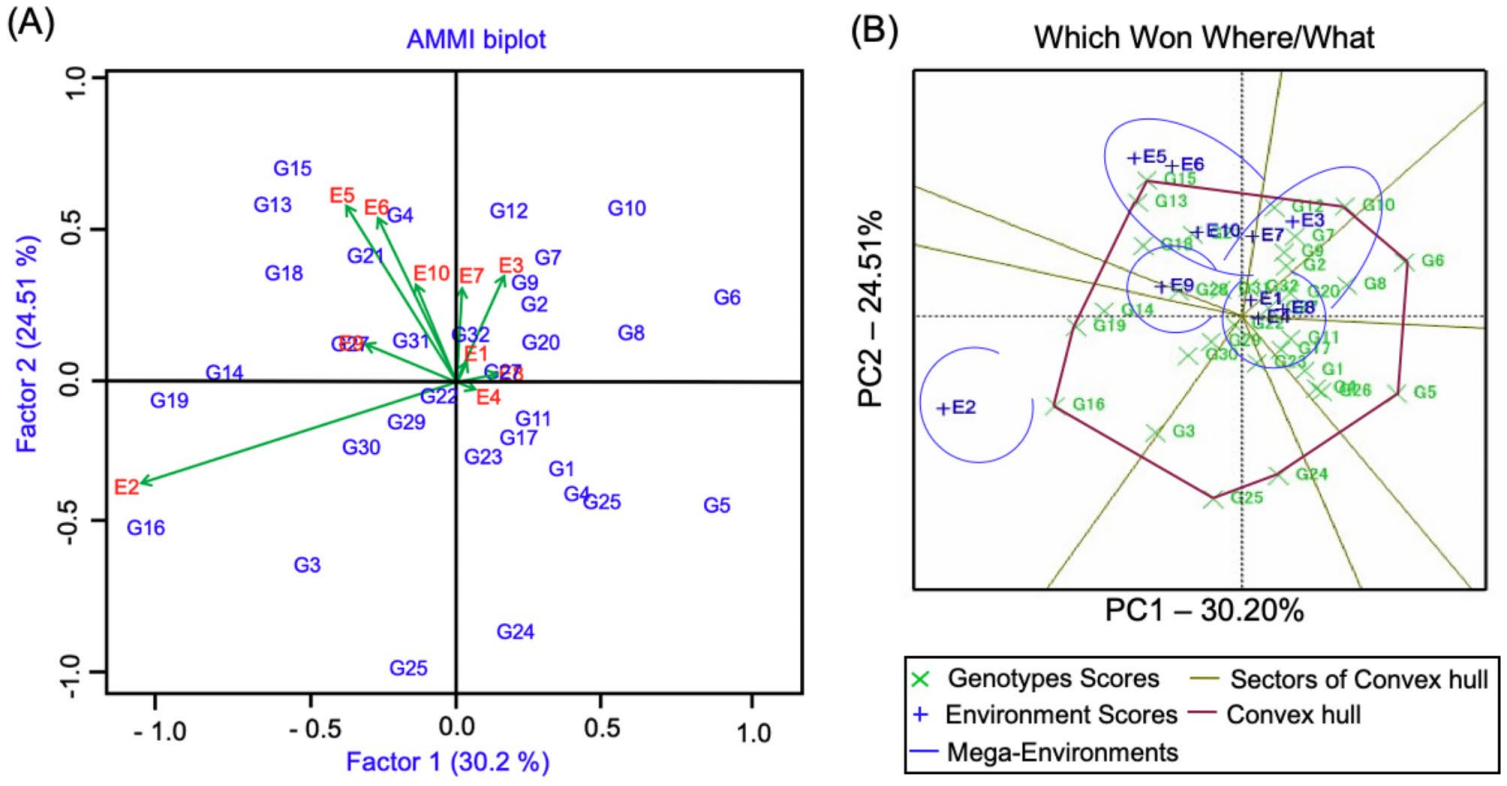
AMMI biplot showing GE interactions for 32 barley genotypes evaluated across 10 test environments. (**B**) A polygon view pattern ‘which-one-where’ of GE interaction for the barley genotypes studied

To identify ideal genotypes for multi-environment performance, the AMMI biplot demonstrated genotype discrimination across different environments (Fig. 3A). Genotypes G10, G6, and G8 showed the highest IPCA1 values, while G22 and G23 had IPCA1 values closest to zero. Environments E2, E5, and E6 recorded the highest IPCA1 values (Fig. 3A) and yield productivity (Table 4), indicating their significant impact on genotype discrimination. Conversely, environments E1 and E4, with the lowest grain yield, had IPCA1 values closest to zero, showing minimal impact on GEI (Table 4). E3 and E7 greatly contributed to GEI, as indicated by their long vectors (Table 5). Environments with acute angles between their vectors were strongly associated with genotypes ranked in the same direction, while those with obtuse angles were least associated, and right angles indicated no association. Positive associations were observed between environments E9 and E5, and E5 and E6, which were negatively correlated with E3 and E7. Genotypes G10, G6, and G8 interacted positively with E3 and E7 but negatively with E2 (Fig. 3A). Several genotypes, such as G13, G15, G18, and G21, exhibited general adaptation with high grain yield (Table 4), while most exhibited specific adaptability.

**Table 5:** Grouping of environments using the dominant yielding genotypes and recommending the first four barley genotypes for the test environment using the AMMI model

Environments	Mean (tons ha^− 1^)		The first four AMMI-recommended genotypes			
		IPCA Score	1	2	3	4
E3	4.50	1.0396	G10	G6	G2	G7
E7	3.29	0.644	G10	G2	G17	G9
E8	2.57	0.513	G10	G17	G2	G9
E6	5.96	0.2848	G15	G13	G21	G12
E1	0.96	0.2702	G10	G2	G18	G9
E4	1.28	0.2465	G10	G17	G16	G2
E10	5.90	0.1773	G15	G13	G18	G21
E5	9.00	-0.0145	G15	G13	G19	G21
E9	3.32	-0.3694	G16	G14	G18	G19
E2	7.55	-2.7915	G16	G19	G14	G3

Tons ha^− 1^, tons per hectare; IPCA, interaction principal component axis

#### Genotypes recommended by the AMMI model

According to the AMMI results, four promising genotypes were identified for each environment (Table 5). Genotype G10 was ranked as the top genotype in five environments: E3, E7, E8, E1, and E4. Genotype G15 was prominent in three environments: E6, E10, and E5 (Table 4). Genotype G16 excelled in E9 and E2, while G13 dominated in E6, E10, and E5, ranking among the top four in three environments. G18 was dominant in E1, E10, and E9, ranking among the top four in four environments. These findings suggest that the AMMI model effectively identifies well-adapted genotypes for specific environments.

### GGE biplot analysis

#### The winning genotypes for the studied environments

The AMMI’s residual mean square was highly significant (Table 3), qualifying the creation of a GGE biplot to visualize the “which-won-where” patterns of environments and genotypes, as well as the environment’s discriminative ability and representativeness. Based on the “which-wins-where” pattern, environments and genotypes were divided into five and six groups, respectively (Fig. 3B). A polygon on the GGE biplot was created by connecting the vertex genotypes with straight lines, while the other genotypes were positioned inside the polygon.

Vertex genotypes G15, G10, G6, G5, G25, G16, and G19 were identified as either the best or poorest performers in one or more environments due to their greatest distance from the origin of the biplot. Genotypes inside the polygon and closer to the origin are widely adapted with minimal response to environmental differences [42, 43]. The responsive genotype has either the poorest or the best performance in a single or all environments [44]. Genotypes G13, G15, and G18 exhibited high yields with overall adaptation, while G24 was the lowest-yielding genotype among the vertex genotypes.

Environments falling inside the sectors of vertex genotypes G13 and G15 indicated these genotypes were among the best in all studied environments. The GGE biplot also identified five mega-environments (ME1, ME2, ME3, ME4, ME5). Testing environments E5, E6, and E10 were in ME1; E3 and E7 were in ME2; E1, E4, and E8 were in ME3; E9 was in ME4; and E2 was in ME5. The vertex genotypes in each sector were the best performers in their respective environments, with G13, G15, and G18 being the high-yielding genotypes in ME1, ME2, and ME3, respectively. The GGE biplot polygon view effectively identifies winning genotypes by displaying interaction patterns between genotypes and environments.

#### Selection for yield and stability performance

**Fig. 4:**
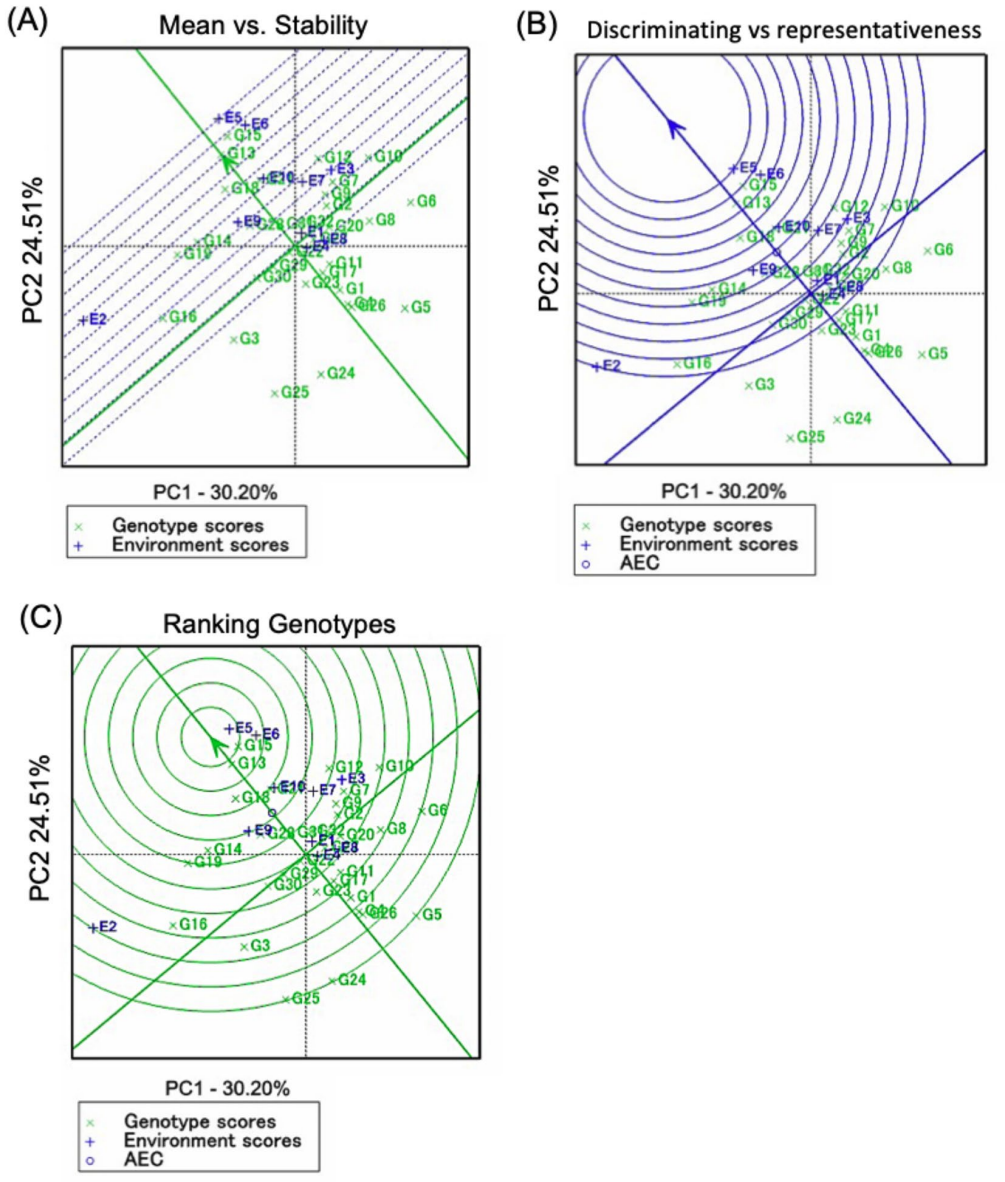
GGE biplot analysis for the yield performance of 32 barley genotypes evaluated across 10 test environments. (**A**) The mean vs. stability view for the grain yield and the environment. (**B**) Discriminating power and representativeness of the test environments. (**C**) Ranking of 32 barley genotypes relative to the ideal genotype

The GGE biplot (Fig. 4A) ranked genotypes by mean yield performance along the ‘average tester coordination’ (ATC) abscissa. G13 had the highest mean yield, followed by G18, G15, G14, G16, G21, G19, G10, G28, G32, G9, G2, G7, G12, G31, and G17, all exceeding the mean yield of 4.43 tons ha⁻¹ (Table 3). In contrast, G1, G4, G17, G13, G23, G26, and G27 had the lowest contributions to GEI. The yield and stability of G13, G15, G18, and G32 were the highest.

#### Assessment of environments studied and genotype adaptation

The environment’s discrimination ability is relative to the length of its vector, with longer vectors indicating a greater ability to differentiate genotypes [42]. In our study, environments E2, E5, and E6 showed the highest discrimination power, highlighting significant genotype differences, whereas E1, E8, and E4 had the lowest (Fig. 4A). Representativeness is determined by the angle between the average environment coordinate (AEC) axis and an environment’s vector; smaller angles indicate stronger representativeness [25]. Environments E9, E10, E6, and E5 exhibited strong representativeness (Fig. 4B).

An ideal environment has both high discrimination power and representativeness. E5 was identified as the ideal environment, excelling in both aspects (Figs. 3A and 4B). Nonrepresentative environments like E3 are useful for selecting genotypes adapted to specific conditions or discarding unstable ones within a single mega-environment. Genotypes G13, G15, G18, and G21 were most adapted to ideal environments, with G15, G13, and G32 emerging as the ideal genotypes due to their high yield and stability (Fig. 4C).

#### Assessment of genotypes according to stability indices

A total of 22 univariate and multivariate models were used to investigate the stability performance of the genotypes studied (Tables 6 and 7). According to grain yield (GY) as a key parameter for genotype assessment, G13, G18, and G15 exhibited the highest mean performance across environments, while G24, G25, and G1 had the lowest grain yield. These outcomes were confirmed graphically by the GGE biplot method (Fig. 4B; Table 6).

**Table 6:** The mean yield stability values based on different parametric stability statistics for 32 barley genotypes grown in 10 test environments

			Deviation mean squares				Join regression				Tai		
GEN.	GY	CV	b_i_	S^2^_di_	*R* ^2^	σ_i_^2^	B_i_	D_ji_	W^2^_i_	S_xi_^2^	α_i_	λ_i_	D_i_^2^
G1	3.96	61.86	0.882	0.176	0.965	0.30	-0.118	0.236	2.81	6.00	-0.118	3.614	8.30
G2	4.61	60.41	0.980	1.026	0.881	1.00	-0.020	1.086	8.72	8.11	-0.020	16.669	8.70
G3	4.20	75.60	1.089	1.362	0.875	1.38	0.089	1.422	11.90	10.08	0.089	21.819	8.85
G4	4.13	54.54	0.765	0.755	0.857	1.18	-0.235	0.814	10.22	5.08	-0.236	12.471	8.57
G5	3.81	51.03	0.654	0.610	0.842	1.55	-0.346	0.670	13.36	3.78	-0.347	10.230	8.50
G6	4.16	61.24	0.781	2.153	0.697	2.45	-0.220	2.213	20.93	6.50	-0.220	33.937	9.20
G7	4.71	53.72	0.873	0.758	0.886	0.87	-0.127	0.818	7.62	6.40	-0.127	12.546	8.57
G8	4.15	64.67	0.924	0.909	0.881	0.93	-0.076	0.968	8.14	7.21	-0.077	14.858	8.64
G9	4.73	55.01	0.925	0.407	0.939	0.45	-0.075	0.467	4.11	6.78	-0.075	7.168	8.41
G10	4.85	56.50	0.902	1.575	0.806	1.59	-0.098	1.635	13.72	7.50	-0.099	25.080	8.95
G11	4.19	63.07	0.931	0.553	0.922	0.58	-0.069	0.613	5.22	6.99	-0.069	9.398	8.48
G12	4.60	65.18	1.064	1.064	0.894	1.06	0.064	1.124	9.26	9.42	0.064	17.242	8.71
G13	4.83	70.65	1.311	0.341	0.973	1.11	0.311	0.400	9.68	13.14	0.312	6.098	8.38
G14	5.06	64.08	1.119	1.280	0.887	1.35	0.119	1.340	11.67	10.51	0.119	20.557	8.81
G15	5.78	73.35	1.323	0.970	0.934	1.77	0.323	1.030	15.21	13.93	0.324	15.757	8.67
G16	4.74	71.49	1.131	3.295	0.761	3.28	0.131	3.354	27.98	12.50	0.132	51.468	9.68
G17	4.52	49.62	0.757	0.805	0.847	1.25	-0.243	0.865	10.86	5.04	-0.244	13.249	8.59
G18	5.10	64.51	1.188	0.286	0.972	0.57	0.188	0.346	5.14	10.81	0.189	5.289	8.35
G19	4.86	74.90	1.264	1.471	0.897	1.97	0.264	1.531	16.91	13.24	0.265	23.459	8.90
G20	4.26	62.39	0.929	0.677	0.907	0.70	-0.071	0.737	6.24	7.07	-0.071	11.311	8.54
G21	4.89	65.62	1.157	0.334	0.966	0.54	0.157	0.394	4.81	10.31	0.158	6.033	8.37
G22	4.46	59.96	0.955	0.365	0.947	0.38	-0.045	0.424	3.53	7.16	-0.045	6.509	8.39
G23	4.11	73.43	1.080	0.453	0.950	0.50	0.080	0.513	4.53	9.13	0.080	7.867	8.43
G24	3.66	66.32	0.850	0.521	0.912	0.69	-0.150	0.580	6.15	5.89	-0.150	8.897	8.46
G25	3.78	80.24	0.983	2.221	0.780	2.13	-0.017	2.281	18.27	9.22	-0.017	35.006	9.23
G26	3.90	68.84	0.901	1.266	0.837	1.30	-0.099	1.326	11.26	7.22	-0.099	20.338	8.81
G27	4.16	65.30	1.001	0.285	0.961	0.29	0.001	0.345	2.76	7.76	0.001	5.288	8.35
G28	4.79	67.86	1.158	0.596	0.945	0.78	0.158	0.656	6.91	10.55	0.158	10.049	8.50
G29	4.47	66.91	1.070	0.446	0.950	0.48	0.070	0.506	4.37	8.96	0.070	7.756	8.43
G30	4.34	67.99	1.053	0.471	0.946	0.49	0.053	0.531	4.43	8.72	0.053	8.141	8.44
G31	4.53	64.73	1.051	0.380	0.955	0.40	0.051	0.440	3.69	8.60	0.051	6.752	8.39
G32	4.75	55.13	0.951	0.108	0.978	0.14	-0.050	0.168	1.51	6.87	-0.050	2.577	8.26

Gen.; Genotype, GY; Grain yield (tons ha^− 1^), CV; Coefficient of variability, b_i_; Linear regression coefficients, S^2^_di_; Deviations from regression, R^2^; Coefficient of determination, σ_**i**_^**2**^; Stability variance, *β*_*i*_; Perkins and Jinks’s stability parameters, W^2^_i_; Wricke ecovalence, S_xi_^2^; Roemer’s environmental variance, λ_i_ and *α*_*i*_; Tai’s stability statistics, D_i_^2^; Hanson genotypic stability

**Table 7:** The mean yield stability values based on different nonparametric stability statistics for 32 barley genotypes grown in 10 test environments

		Nassar and Huehn		Thennarasu				Ketata’s plotting rank		
GEN.	ASV	S^I 3^	S^I 6^	NP ^I (3)^	NP ^I (4)^	RSM	YSI	δr	kr	TOP
G1	0.373	11.06	1.89	0.29	1.48	31	-4	5.22	22.20	0
G2	1.894	50.61	4.94	0.57	2.05	30	13	9.43	15.80	40
G3	0.349	32.38	3.29	0.40	1.51	47	1	8.65	20.80	10
G4	1.151	53.71	4.81	0.58	1.83	47	-3	10.42	18.20	30
G5	0.920	49.95	4.30	0.59	1.72	56	-6	10.35	19.30	20
G6	1.421	47.64	4.57	0.57	1.87	55	-3	9.57	17.30	20
G7	1.328	52.51	5.73	0.71	2.77	29	9	8.30	11.80	60
G8	2.579	34.39	3.48	0.44	1.56	42	-4	8.87	20.60	20
G9	0.864	48.42	5.87	0.62	2.62	17	8	8.17	12.40	60
G10	0.639	67.97	5.95	0.77	2.75	35	10	9.48	11.90	40
G11	1.970	43.67	4.33	0.54	1.81	35	-4	9.35	18.00	30
G12	0.905	68.67	6.06	0.67	2.30	33	6	10.26	13.80	50
G13	0.695	60.52	5.92	0.83	2.43	21	14	9.17	12.50	50
G14	0.506	85.36	7.19	0.89	2.57	28	11	10.71	12.10	50
G15	0.179	39.91	4.38	0.81	2.49	31	11	7.32	12.10	30
G16	0.188	81.71	6.55	0.72	1.92	37	10	12.28	16.60	40
G17	0.917	74.02	6.83	0.72	2.39	38	4	10.69	13.90	50
G18	1.696	86.97	7.39	0.76	2.62	13	9	10.95	12.40	60
G19	0.509	70.86	6.55	0.83	2.17	36	7	10.46	13.90	50
G20	0.395	31.80	3.45	0.46	1.85	35	-4	7.82	17.30	20
G21	0.581	51.00	5.28	0.75	2.67	16	6	8.21	11.90	50
G22	0.503	36.81	3.92	0.45	2.07	22	-2	8.04	15.80	20
G23	1.724	24.67	3.15	0.34	1.40	36	-5	7.80	22.20	0
G24	0.876	29.02	3.00	0.41	1.39	45	-8	8.67	23.30	10
G25	0.392	34.56	3.25	0.45	1.42	61	-8	9.17	21.90	10
G26	0.331	55.29	4.83	0.56	1.68	52	-8	10.77	18.90	30
G27	0.813	18.69	2.46	0.31	1.75	22	-8	6.17	18.30	10
G28	1.386	47.63	5.20	0.60	2.13	24	-1	8.82	14.70	50
G29	2.091	26.73	2.98	0.43	1.93	24	-7	6.98	16.40	20
G30	1.119	30.81	3.35	0.39	1.67	27	-8	8.11	19.20	20
G31	1.292	36.65	4.29	0.53	2.08	20	-5	7.92	15.40	40
G32	0.908	23.47	3.70	0.42	2.57	11	3	5.76	12.70	40

Gen.; Genotype, GY; grain yield (tons ha^− 1^), ASV; AMMI stability value, RSM; Kang’s rank-sum, YSI; Yield stability index, δ r, and kr; Ketata’s plotting mean rank, TOP; Fox’s TOP-rank stability parameter

According to the parametric stability statistics, the coefficient of variability (CV) revealed that G7, G5, and G17 were the most stable with low CV and high GY, while G24, G26, and G25 were unstable (Table 6). Eberhart & Russell’s deviation mean squares showed that G27, G2, and G32 were adaptable to favorable conditions (b_i_ ≥ 1), whereas G15, G13, and G19 were suited for unfavorable conditions (bi < 1). G32, G1, and G27 had the lowest variance in deviation from regression (S^2^_di_ ≤ 0), showing the highest stability. The coefficient of determination (R^2^) indicates that G32, G13, and G18 had high R^2^ values, indicating stability, while G6 and G16 were unstable. Wricke’s Ecovalence (W^2^_i_) and Shukla’s stability variance (σ_i_^2^) showed that G32, G27, and G1 were stable, whereas G25, G6, and G16 were unstable. Perkins and Jinks’s stability model revealed that G27, G25, and G2 had the lowest regression coefficients (*β*_*i*_), indicating stability, while G32, G1, and G27 had minimal nonlinear sensitivity to environmental changes (*D*_*ji*_). Roemer’s environmental variance (S_xi_^2^) indicates that G5, G17, and G4 exhibited minimal variance, indicating high stability. Tai Stability Statistics (λ and α) showed that G32, G1, and G27 were perfectly stable (α = -1, λ close to 1), while G15, G13, and G19 showed average stability. Hanson stability (D_i_^2^) showed that G32, G1, and G27 had the lowest D_i_^2^ values, showing high stability.

**Fig. 5:**
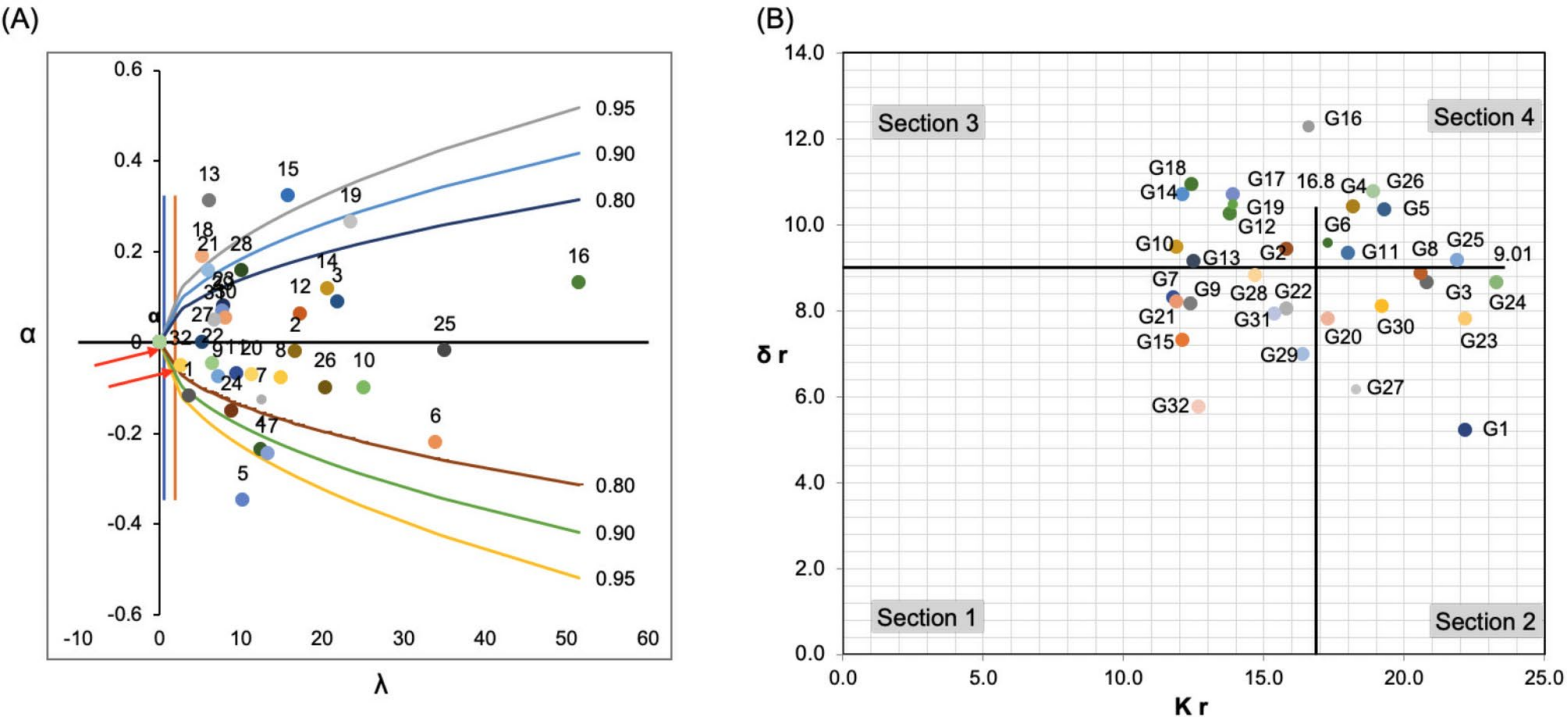
(**A**) The stability of the genotypes was determined based on Tai’s [18] model. (**B**) A plot of the rank standard deviation (δr) against the rank (kr) for genotypes across 10 environments

Furthermore, for the nonparametric stability models, the AMMI Stability Value (ASV) indicates that G15, G16, and G26 were the best-ranked genotypes, while G8, G11, and G29 were unstable (Table 7). Yield stability index (YSI) showed that G13, G2, G14, and G15 combined high yield performance and stability (Fig. 4C). Nassar and Huhn’s model revealed that G32, G1, and G27 were the most stable genotypes with low SI ^3^ and SI ^4^ measures. Thennarasu model exhibited that G32, G1, and G27 displayed the lowest values for NPI ^(3)^ and NPI ^(4)^, indicating stability. Ketata et al. [28], ranking method showed that G32, G15, G7, and G9 in Sect. 1 had the highest mean and stability performance, while G27 was stable in Sect. 2 (Fig. 5B). TOP-Rank stability parameter displayed that G7, G9, and G18 were the most stable, ranking in the top third of genotypes with high mean yields of 4.71, 4.72, and 4.86 tons ha-1, respectively (Table 4).

Overall, G32, G27, and G1 were the most stable genotypes, excelling in 15, 15, and 11 stability statistics out of the 22 models used. These genotypes are promising candidates for barley breeding programs to enhance production and stability. Additionally, G15, followed by G13, G7, and G9, were identified as the most stable genotypes based on multivariate analysis alone.

#### Selecting the best stability statistics

**Fig. 6:**
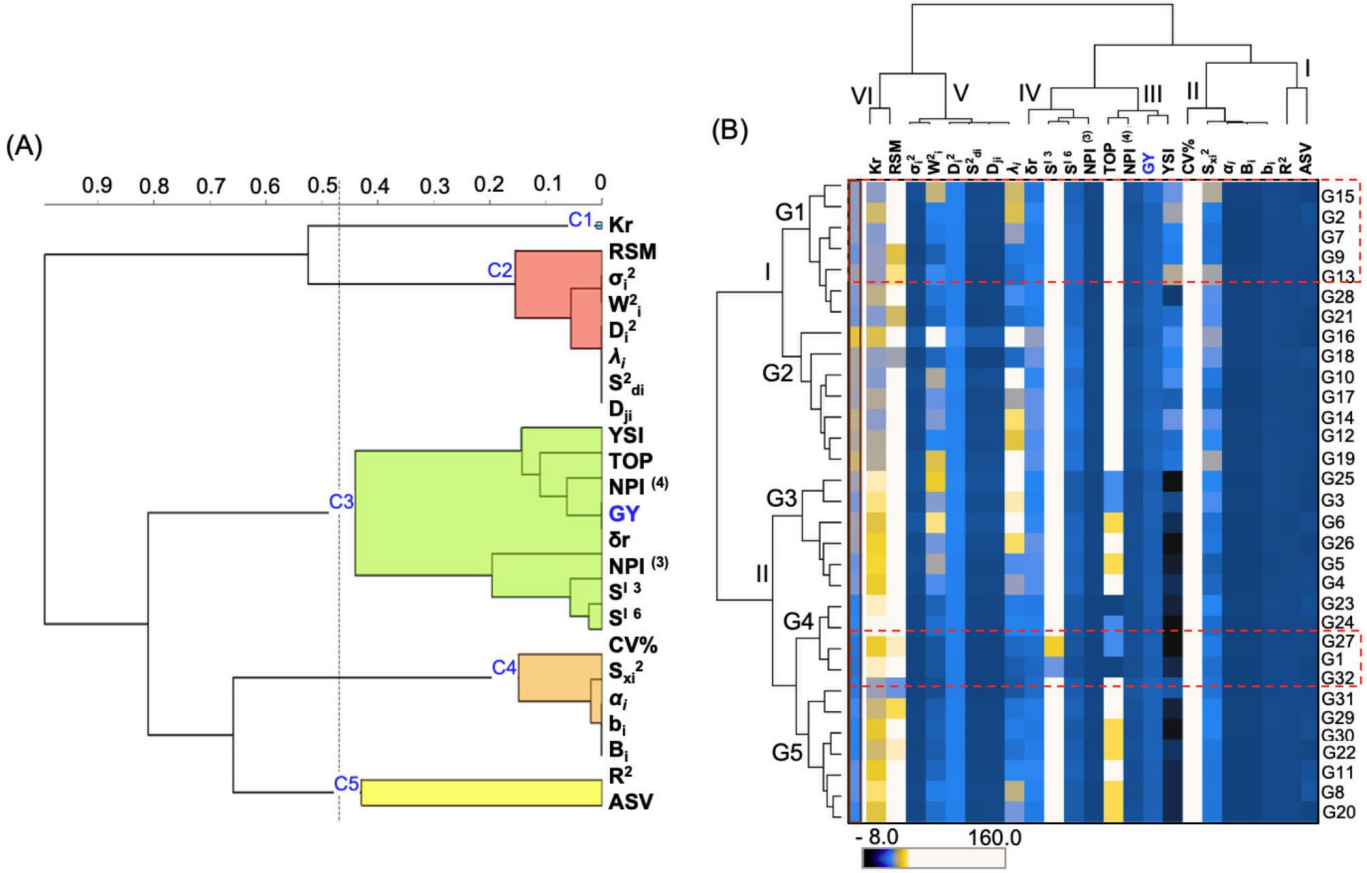
The best stability statistics were selected. **(A)** Hierarchical clustering is based on Ward’s method for grouping the stability parameters. **(B)** A correlation heatmap displays the correlation between genotypes and stability variables to identify the best stability parameters for the simultaneous selection of high yield and stable performance

To determine the most effective stability parameters for selecting high-yield, stable genotypes, a hierarchical cluster analysis was conducted using a rank correlation matrix [41]. The analysis divided the 22 stability measures into five clusters (Fig. 6A). Cluster 1 (C1) featured Ketata’s plotting mean rank (Kr), focusing on genotype stability. Cluster 2 (C2) grouped measures such as RSM, σ_i_^2^, W^2^_i,_ D_i_^2^, λ_i_, S^2^_di_ and D_ji_, indicating a comprehensive view of genotype stability. Cluster 3 (C3) included nonparametric stability statistics like YSI, TOP, NP ^I (4)^, δr, NP ^I (3)^, S^I 3^ and S^I 6^, which correlate with yield and stability. Cluster 4 (C4) contained CV, S_xi_^2^, α_i_, b_i_ and B_i_, reflecting different aspects of stability. Cluster 5 (C5) encompassed ASV and R2, related to performance and stability.

A pattern map based on squared Euclidean distance categorized 32 barley genotypes into five distinct classes, revealing genetic diversity (Fig. 6B). Genotypes G32, G27, and G1 appeared in the most stable Classes G4 and G5 (Table 6). Groups were also formed based on the stability parameters: Group I (R^2^, ASV), Group II (CV, bi, Bi, αi, Sxi2), Group III (TOP, NPI (4), YSI, GY), Group IV (NPI (3), SI 6, SI 3, δr), Group V (σ_i_^2^, W^2^_i_, D_i_^2^, S^2^_di_, D_ji_, and *λ*_*i*_), and Group VI (K_r_, RSM). Using diverse stability parameters from these groups can reduce overlap and enhance the selection of stable genotypes.

#### Associations between the stability parameters

**Fig. 7:**
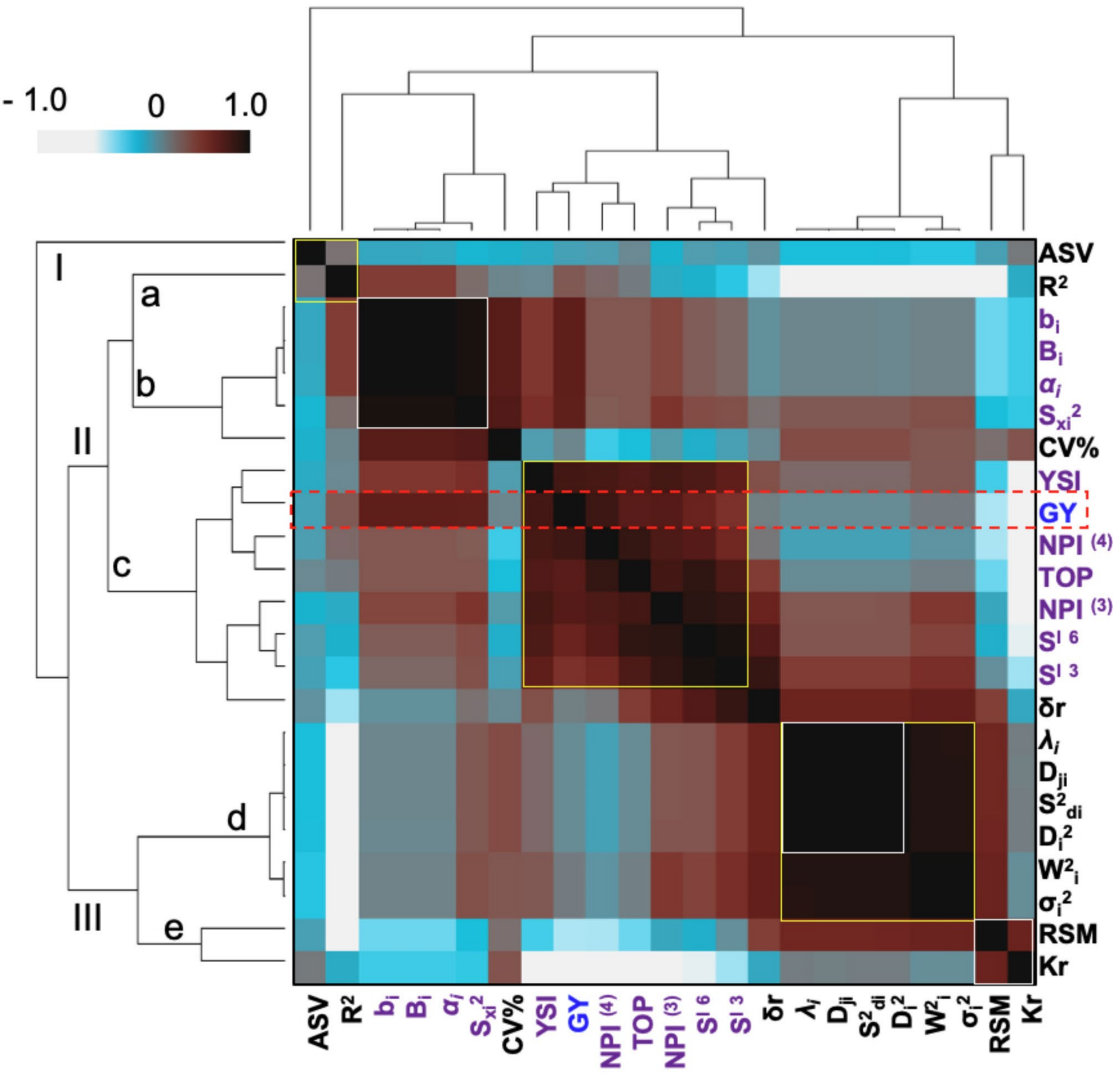
Hierarchical clustering between the 22 stability parameters. The heatmap colors indicate the Spearman correlation coefficient among the stability parameters. A darker color indicates a greater correlation. The number of Latina indicates the subgroup

To explore the associations between parametric and nonparametric stability models, we used Spearman’s correlation matrix for graphical evaluation (Fig. 7 and Supplementary File 4). The analysis revealed that stability parameters clustered into three main groups (I-III) and five subgroups (a-e) (Fig. 7). Grain yield (GY) showed a strong positive correlation (*P* < 0.01) with univariate stability statistics such as b_i_, *α*_*i*_, B_i_, and S_xi_^2^, which are grouped in subgroup (b) of cluster II (Fig. 7). Additionally, GY was positively correlated with multivariate measures including SI ^6^, NPI ^(3)^, NPI ^(4)^, YSI, and TOP, which are in subgroup (c) of cluster II (Fig. 7). Conversely, GY was negatively correlated (*P* < 0.01) with RSM and kr. The coefficient of regression (b_i_) was significantly associated with B_i_, S_xi_^2^ and *α*_*i*_. Deviation from regression (S^2^_di_) showed a significant positive correlation with σ_i_^2^, D_ji_, W^2^_i_, λ_i_ and D_i_^2^. Notably, σ_i_^2^ was positively correlated with D_ji_ and W^2^_i_, while W^2^_i_ was significantly associated with λ_i_ and D_i_^2^. S_xi_^2^ had a positive correlation with αi, and λi was significantly related to D_i_^2^. SI ^3^ and SI ^6^ were positively correlated with NPI ^(3)^, NPI ^(4)^, and TOP.

## Discussion

The identification of high-yield and stable cultivars across a broad range of environmental conditions is the ultimate objective of breeders because it is more complex due to the presence of GEI [45]. The GEI causes variation in genotype responses to different environmental conditions and causes bias in the selection procedure [46]. There are two approaches for improving genotypes with a minimum G x E interaction. First, the stratification or subclasses of heterogeneous regions were divided into smaller, additional homogeneous subareas to improve genotypes for specific sub regions. The second approach is based on decreasing the impact of G × E, which includes selecting genotypes with greater stability through a broad range of conditions to produce a good prediction of their performance [15, 18]. In this study, we evaluated 32 barley genotypes in 10 different environments and suggested that genotype quantification by environmental interaction, mega-environmental classification, environmental characterization, and consequential selection of genotypes according to stability and yield performance might be applicable. Several models that use G x E interactions have been developed to accelerate the characterization of stable genotypes and as a selection index jointly with the yield performance of the genotypes. Therefore, genotypes with high or low yields and lower yield variation in different environments are considered stable. This may be well-founded as a static or biological concept of stability, which might not be preferred to most breeders, who prefer the dynamic (agronomical) concept of stability, which refers to high-yield performance genotypes with a potential response to agronomic inputs or improved environmental conditions [12]. We found that the environment greatly affected approximately 81.3% of the total variation. This value is close to the 81.2% obtained from a study by Mohammadi et al. [47], wherein 24 durum wheat genotypes were evaluated across 21 environments. Furthermore, the variance between the highest (E5) and lowest (E1) environments was 8.41 tons ha^− 1^ for yield performance (Table 6), which is greater than the 8.5 times that of the lowest-yielding environment. In addition, GEIs had a highly significant impact of approximately five times (15.7%) greater than the effect of genotype (2.9%), causing significant GEIs and great variations in the ranking of genotypes [48]. The effect of high GEIs could also impact the prediction of a genotype’s response to the environment and therefore decrease heritability and untimely selection procedures [6, 46, 49]. To study the stability, model-based deviation means squares such as those used by Eberhart and Russell [15], were used in this study because yield variation for each genotype can be split into unpredictable (variance deviation) and predictable (regression) components. A higher yield with a regression coefficient of b_i_ ≥ 1 was detected for the G32, G27 and G1 genotypes, demonstrating relatively greater performance than the overall mean. However, based on graphical stability models such as the yield stability index (YSI) and AMMI stability value (ASV), G14 was the most stable genotype for different environments, followed by G15 and G13.

The evaluation of barley genotypes through MET analysis revealed significant variation in both yield and stability performance. Consequently, MET could be effectively utilized to enhance breeding approaches aimed at improving both yield and stability. To estimate genotypic stability, genotypes that were demonstrated to be stable for more than half of the stability parameters with higher or equal mean performance to the grand mean yield were selected as promising genotypes [49, 50]. In our study, the G32, G27, and G1 genotypes were found to be desirable and stable. When genotypes exhibit stable yield performance across a wide range of ecological conditions, their use and wide adaptation to cultivation could increase [49, 50]. Furthermore, the analysis shows the presence of GEIs for yield through environments that include locations with differences in winter precipitation and temperature, leading to a mega-environmental association. The importance of GEIs for yield performance in barley was detected in previous studies [49, 50]. The results also showed that for every year, the locations fell into different sets, and the location combination patterns differed over the years. The first two IPCAs explained 54.4% of the total variance as a result of the G and G x L (genotype by location) effects each year and the average of the two years (Fig. 3), suggesting the presence of mega-environments. Based on our results, we discovered five mega-environments with their winning genotypes, which highlights the particular adaptations of a genotype to its related mega-environment and positive utilization of GEIs [47, 48]. Our results also indicated that some genotypes were better adapted to some environments in Egypt than others.

GGE biplots offer chances to identify differences among environments with different properties according to the genotype’s performance in these environments. Among these advantages is a ‘which-won-where’ polygon view of the genotypes [48, 51]. The polygon vertices are the genotype signs identified farthest from the biplot origin in numerous directions, such that all genotype signs are bounded inside the polygon. The vertex genotype for each region is the one with the highest yield for the environments that are bounded inside that zone. An additional significant feature of the biplot is that it displays the environmental categories, which suggests the potential presence of different mega-environments [38, 52]. An ideal evaluation environment must be more representative of all environments and discriminate among genotypes [26, 53, 54]. The study locations may be divided into three types according to the discriminating vs. representativeness of the GGE biplot power view. Type I environments, in which short vectors provide minimal evidence on genotypes, are not considered study environments. Type II environments, which have small angles and elongated vectors for average environment coordinates (AECs), are therefore ideal environments for detecting elite genotypes. Type III environments, which contain large angles and long vectors with average AECs, thus cannot be considered for selecting elite genotypes; however, such environments would be helpful for removing unstable genotypes [9]. The results indicated that between the ten studied locations, E2, E5, and E6 had elevated representativeness and discriminating power and thus were the best locations for selecting elite genotypes (Fig. 4B). E1 was a Type III environment and/or an unnecessary environment that would be avoided to decrease the yield evaluation costs [55].

Hierarchical cluster analysis indicated that the G13, G15, G7 and G9 genotypes were separated into high-yield and stable-performance genotypes in the genotypic G1 class (Fig. 6B). This result could be consistent with Yan and Kang’s view that an ideal genotype has one with high and stable performance across different environments [54]. We found that both the numerical and graphical models revealed a related outline for the identification of ideal genotypes. For example, univariate and multivariate measures identified numerous genotypes, such as G5, G7, G13, G15, G18, G27 and G32, as highly stable in comparison with the others. Similarly, GGE biplots classified several of those genotypes, such as G13, G15, and G18, which are vertex genotypes and are promising in the environment bounded within their respective sectors of the GGE biplot polygon view. Similarly, Elakhdar et al., investigated the GGE biplots and both numerical methods to identify stable barley genotypes across different areas of Egypt [6]. The authors described the relative influence of both approaches on the identification of ideal genotypes. The relative contributions of stability and yield performance from the GGE biplot method in our study were similar to those in previous studies on barley [48, 56], durum wheat [47, 50], maize [55], and cowpea [57].

The univariate and multivariate stability models provided significant characteristics in several statistical assumptions, such as the normal distribution; nonetheless [32], they cannot be implemented correctly if these assumptions are violated. Parametric statistics for the mean square of the significance might be more complex than the fundamental assumptions. Therefore, it is wise to explore alternative methods that are highly robust to differences from usual assumptions such as nonparametric statistics [24, 32]. Hierarchical clustering distinguishes methods based on relatively associated stability parameters within each cluster (Fig. 7). This suggests that selection for yield performance based on these parameters is ideal. The GY had a remarkable variation and positive correlations with the b_i_, *α*_*i*_, B_i_, S_xi_^2^, NP^I (3)^, S^I 6^, S^I 3^, TOP, NP^I (4)^ and YSI clustering group II (c) statistics; therefore, selection depending on these stability approaches is ideal because it is associated with the dynamic concept of stability [12]. Similar results were obtained for barley [48, 56] and durum wheat [47, 50]. A close correlation has been observed between GY and YSI [58] and between GY and TOP and between GY and Bi [48]. Thus, such parameters could stipulate a degree of stability in the dynamic concept. In particular, these methods could be used for selecting genotypes with favorable growing conditions adapted to the environment [59]. Furthermore, subgroup (d) of cluster III (Fig. 7) was composed of S^2^_di_, which was strongly correlated with D_ji_ [17], λ_i_ [18], and D_i_^2^ [23]; σ_i_^2^, which was strongly correlated with W^2^_i_ [19]; and D_ji,_ which was strongly correlated with λ_i_ [18] and D_i_^2^ [23]; this subgroup was used for the analysis of stability based on the static concept. Notably, not all measures were significantly associated with yield performance. Consequently, these measures permit the identification of genotypes with unfavorable growing conditions adapted to the environment. Our results indicate that the associated measures are similar to genotype ranking and might be utilized as alternatives for stability analysis, as they rank genotypes in a related way under diverse cultivating conditions, so one of them might be utilized for stability analysis [50].

## Conclusions

The purpose of this study was to determine the optimal combination of stability parameters for the assessment of the stability and yield performance of high-yield potential and stable cultivars in different environments. The study was concluded using 32 barley genotypes that were evaluated in 10 environments. Both the univariate and multivariate stability models showed that genotypes G32, G1, and G27 were the most stable genotypes with minimal yield variation across environments. Additionally, G13, followed by G15, G7 and G9, were the most stable genotypes based on multivariate measures only. The findings of this study suggest the use of a combination of stability models with respect to static and dynamic concepts of stability for the selection of “ideal genotypes” of high-yield potential and stable cultivars.

## Electronic supplementary material

Below is the link to the electronic supplementary material.

Supplementary Material 1

Supplementary Material 2

Supplementary Material 3

Supplementary Material 4

## Data Availability

No datasets were generated or analysed during the current study.

## Abbreviations

E1: South Sinai (2021–2022)
E2: Gemmeiza (2021–2022)
E3: New Valley (2021–2022)
E4: Ismailia (2021–2022)
E5: Sakha (2022–2023)
E6: Giza (2022–2023)
E7: New Valley (2022–2023)
E8: Ismailia (2022–2023)
E9: Gemmeiza (2022–2023)
E10: Nubaria (2022–2023)
GEN: Genotype
GY: Grain Yield (tons hay ^− 1^), tons ha^− 1^, tons per hectare
IPCA: Interaction Principal Component Axis
CV: Coefficient of Variability;
b_i_: Linear regression coefficients;
S^2^_di_: Deviations from regression;
R^2^: Coefficient of determination;
σ^2^_i_: Stability variance
>βi: Perkins and Jinks’s stability parameters
W^2^_i_: Wricke Ecovalence
S_xi_^2^: Roemer’s Environmental Variance
λ_i_ and *α*_*i*_: Tai’s stability statistics
D_i_^2^: Hanson genotypic stability
ASV: AMMI Stability Value
RSM: Kang’s Rank-Sum
