# Supplementary material for: Integrating univariate and multivariate stability indices for breeding clime-resilient barley cultivars
Source: BMC Plant Biol. 2025 Jan 18;25:76. doi: 10.1186/s12870-024-05530-6 (PMC11748582; doi:10.1186/s12870-024-05530-6)
Supplement: Supplementary file 2 — Supplementary Material 2 [file 12870_2024_5530_MOESM2_ESM.docx]

**Supplementary file 2**

- - 1. **Estimation of yield stability parameters**

The GGE biplot was estimated according to the symmetrical scaling method, which uses singular value partitioning of the first two principal components analysis (IPCA) [1]. Using the GGE biplot method some graphical analysis comprising genotype evaluation, mega-environment analysis and discriminate power of exam environments against their representativeness ability were investigated. The yield stability index (YSI) of the AMMI model [2] was used to partition the grain yield variation into AMMI stability value (ASV) and AMMI effects [3]. Gollob's F-test [4] was used to preserve the number of significant interactions with IPCA in the model. AMMI and GE biplot analyses were estimated by using GENSTAT software [5]. To estimate the genotype stability, the integration of some univariate, and multivariate stability models was used as follows.

AMMI stability value (ASV) was calculated for each genotype according to the relative contributions of the principal component axis scores (IPCA1 and IPCA2) to the interaction sum of squares.

The AMMI stability value (ASV) as described by Purchase et al. [3] was calculated as follows:

$$ASV=\sqrt{\left[ \frac{SSIPCA}{SSIPCA2}(IPCA1 Score1) \right]^{2}}+ {(IPCA1 Score2)}^{2}$$

Where,

SS = Sum of square of the IPCAs and IPCA1 and IPCA2 are the 1st and 2nd interaction component axes respectively.

Smaller the ASV scores indicate a genotype is more stable across environments, whereas larger the IPCA score either positive or negative, the genotype is more specifically adapted to certain environments.

Yield stability index was also calculated using the sum of the ranking based on yield and ranking based on the AMMI stability value.

$$YSI=RAS+RY$$

where

 RASV is the rank of the genotypes based on the AMMI stability value;

RY is the rank of the genotypes based on yield across environments (RY).

YSI incorporates both mean yield and stability in a single criterion. Low values of both parameters show desirable genotypes with high mean yield and stability [3].

1. **Univariate measurements**

**Regression coefficient:**

The regression coefficient [6] is estimated based on the regression function. Genotype with a low coefficient of regression is considered stable.

Coefficient of regression:

$$bi=1+ \frac{{\sum_{j}(X_{ij}-\overline{X}_{i.}-\overline{X}_{.j}-\overline{X}_{..})}^{2}}{E-1}$$

where *X_ij_* is the observed mean value of genotype *i* (*i* = 1, ..., G) in environment *j* (*j* = 1,…,E), with $\overline{X}_{i.}$ and $\overline{X}_{.j}$indicating minor means of genotype i and environment j, respectively. $\overline{X}_{..}$ indicates the overall mean.

**Deviation means squares:**

A linear correlation among the phenotype and environment and assessed its effect on the trait. Assumed an additional method to find the phenotypic regression (b_i_) of the value y_ij_ in the environment E_j_, as against to phenotypic regression (b_j_) of g_ij_ on E_j_ as suggested by Finlay and Wilkinson, [6]. Analysis of GEIs, using joint regression the interaction sum of squares is divided into two portions: (a) linear regression coefficients (b_i_), (b) the pooled deviations from regression lines (S^2^_di_) according to Eberhart and Russell [7].

Regression coefficients (b_i_):

$$b_{i}=1+ \frac{\Sigma_{i}(X_{ij}-\bar{X}_{i.}-\bar{X}_{.j}+\bar{X}_{..})}{\Sigma_{j}{(X_{.j}-\bar{X}_{..})}^{2}}$$

Deviations from regression (S^2^_di_):

$S_{di}^{2}=\frac{1}{E-2}$ $\left[ \Sigma_{j}{(X_{ij}-\bar{X}_{i.}-\bar{X}_{.j}+\bar{X}_{..})}^{2}-{{(b}_{i}-2)}^{2}\sum{(\bar{X}_{.j}+\bar{X}_{..})}^{2} \right]$

where X_ij_ is *X_ij_* is the observed mean value of genotype *i* (*i* = 1, ..., G) in environment *j* (*j* = 1,..., E), with $\bar{X}_{i.}$ and $\bar{X}_{.j}$ indicating minor means of genotype *i* and environment *j*, respectively. $\bar{X}_{..}$ indicates the overall mean of X. *bi* is the estimation of the coefficient of regression. The genotypes are categorized according to the size of the b^i^ and S^2^_di_ parameters in this model. A genotype is highly adapted to favorable environmental conditions and adapted to unfavorable growing conditions when b_i_ > 1, and b_i_ < 1, respectively. The genotypes expressed an average adaptation to all environments when regression coefficients are equal to one. Genotypes with S^2^_di_ equal to zero could be the most stable, whereas an S^2^_di_ higher than zero would indicate lower stability across the environments.

**Coefficient of determination:**

The coefficient of determination [8] is estimated based on regression function. A genotype with a high coefficient of determination is considered stable.

Coefficient of determination:

$$R^{2}=1- \frac{S_{di}^{2}}{S_{xi}^{2}}$$

where X_ij_ is *X_ij_* is the observed mean value of genotype *i* (*i* = 1, ..., G) in environment *j* (*j* = 1,..., E), with $\bar{X}_{i.}$ and $\bar{X}_{.j}$ indicating minor means of genotype *i* and environment *j*, respectively. $\bar{X}_{..}$ indicates the overall mean of X.

**Join regression analysis:**

Two stability parameters (*βi* and *Dji*) indices were suggested by Perkins and Jinks, [9] as an improvement of Bucio Alian and Hill model [10]. A regression coefficient (*β_i_)* as a linear sensitivity to change in the environments and non-linear sensitivity to the environments change (*D_ji_)*.

$$\beta_{i}=\frac{\Sigma_{j}(Y_{ij}-\bar{Y}_{i.}-\bar{Y}_{.j}+\bar{Y}_{..})(\bar{Y}_{.j}-\bar{Y}_{..})}{\Sigma_{j}{(\bar{Y}_{.j}-\bar{Y}_{..})}^{2}}$$

$$D_{ji}=\frac{\Sigma_{j}\delta_{ij}^{2}}{(s-2)}$$

where *βi* is the linear regression coefficient for the i^th^ genotype and $\delta_{ij}^{2}$ is the division from regression from the i^th^ genotype in the j^th^ environment.

**Coefficient of variation:**

In this model, stability could be estimated through the combination of the mean yield and environmental variance and coefficient of variation according to Francis and Kannenberg, [11]. A genotype with the lowest variance across different environmental conditions, with a minimum CV, and high yield performance is considered the most desirable. This statistic is calculated as follows:

$CV\%=\left( \frac{\sum_{j} \left( Y_{ij}-\overline{Y}_{j.} \right)^{2}}{S-1}/ \overline{Y}_{j.} \right)x 100$, where; S is the standard deviation and Y is the grand mean of the experiment.

**Ecovalence:**

Wricke’s ecovalence is estimated according to a square and sums up the genotype-environment interaction across the environment [12]. A genotype with low ecovalence is considered stable. The greatest stability is when W^2^_i_ = 0.

Ecovalence:

$W_{i}^{2}={\sum( X_{ij}-\overline{X}_{i.} -\overline{X}_{.j} -\overline{X}_{..} )}^{2}$, where X_ij_ is the observed yield response (averaged across experiment explicates), $\overline{X}_{i.}$and $\overline{X}_{.j}$ corresponded to the previous notations, and $\overline{X}_{..}$ is the grand mean.

**Environmental variance:**

Environmental variance ($S_{xi}^{2})$, [13] as described in Becker and Leon [14] shows the variation of every genotype over the studied environments as phenotype stability. A genotype with low variation in different environments is considered to be high stable. This measure is calculated as follows:

$S_{xi}^{2}=\frac{{\sum(X_{ij}-\overline{X}_{i.})}^{2}}{E-1}$, where $X_{ij}$, $\overline{X}_{i.}$ and E correspond to previous notations.

**Stability variance:**

Stability variance (σ_2_^i^) is estimated according to a linear combination of the mean square of the G × E interaction and ecovalence [15]. The lower the stability variance, the more stable the genotype.

$\sigma_{i}^{2}=\frac{t}{\left( s-1 \right)\left( t-2 \right)\left( E-1 \right)}\sum_{j} \left( Y_{ij}-\bar{Y}_{i.} -\bar{Y}_{.j} -\bar{Y}_{..} \right)^{2}$- $\frac{\Sigma_{j}{(X_{ij}-\bar{X}_{i.} -\bar{X}_{.j} -\bar{X}_{..})}^{2}}{t-2}$

where X_ij_ is the observed phenotypic mean value of genotype *i* (*i* = 1,2..., t) in environment *j* (*j* = 1,2..., s), with $\overline{X}$ and $\overline{X}$ denoting marginal means of genotype *i* and environment *j*, respectively. $\overline{X}_{..}$ denotes the overall mean.

**Tai’s stability statistics:**

Two parameters of stability; Tai's alpha (α) and lambda (λ), are mean-variance factors for a pairwise G × E interaction proposed by [16]. The normal division of the GEIs into a sum of squares of the deviation from the regression and regression sum of squares is practicable when the effects of the environment can be estimated without error. The linear response to environmental effects could be estimated with statistic *α_i_*, while the deviation from the linear response could be estimated with statistic λ_i_. A stable genotype would have an *α_i_* = -1 and λ_i_ = 1. Conversely, a genotype with average stability has *α_i_* = 0 and *λ_i_* =1, and the value (α > 0, λ = 1) refers to the below average stability.

**Genotypic stability:**

The genotypic stability (D_i_^2^) [17], is estimated according to a linear regression purpose like Wricke’s ecovalence stability coefficient [12] with considering the regression. Consequently, the lower the genotypic stability the more stable the genotype.

$$D_{i}^{2}={\sum_{j}( X_{ij}-\overline{X}_{i.}-b_{min} \overline{X}_{.j}-b_{min}-\overline{X}_{..})}^{2}$$

where X_ij_ is the observed phenotypic mean value of genotype *i* (*i* = 1, ..., G) in environment *j* (*j* = 1,…, E), with $\overline{X}$ and $\overline{X}$ denoting marginal means of genotype *i* and environment *j*, respectively. $\overline{X}..$ denote the overall mean of X. b_min_ is the minimum value of the regression coefficient over all environments.

1. **Multivariate measurements**

The multivariate stability parameter measure includes four rank-based nonparametric stability methods

**Variance of rank**

Nassar and Huhn’s nonparametric stability comprising, the mean of the absolute rank variances of a genotype across environments SI^(1)^, (2) variance between the ranks across the environments SI^(2)^, (3) the sum of absolute deviations for all genotype relative to the mean of ranks SI^(3)^ and (4) rank sum of squares for all genotype relative to the mean of ranks SI^(6)^ [18]. A genotype with a low variation of the ranks is considered stable.

$$S_{i}^{\left( 3 \right)}=\frac{\sum_{j=1}^{n} {(r}_{ij-}\bar{r_{i.}})}{\bar{r_{i.}}}$$

$$S_{i}^{(6)}=\frac{\sum_{j=1}^{n} \left| r_{ij-r_{i.}} \right|}{\bar{r_{i.}}}$$

where r_ij_ is the range of the i^th^ genotype in the j^th^ environment and$\bar{r_{i.}}$: as the mean rank across all environments for the *i_th_* genotype.

Thennarasu's nonparametric stability analysis considers adjusted ranks of genotypes within each test environment. Four stability based on ranks of adjusted means of the genotypes in each environment measures of nonparametric statistics, NP_I_^(1)^, NP_I_^(2)^, NP_I_^(3)^ and NP_I_^(4)^ [19] and defined stable genotypes as those whose position in relation to the others remained unaltered in the set of environments assessed. The formulae to compute these are given below:

$${NP}_{3}=\frac{\sqrt{\sum\frac{\left( r_{ij}^{*}-\bar{r}_{i.}^{*} \right)^{2}}{N}}}{\bar{r}_{i.}}$$

${NP}_{4}= \frac{2}{N(N-1)\left[ \sum_{j-1}^{n-1} \sum_{j^{'}-j+1}^{n} \left| r_{ij}^{*}- r_{ij'}^{*} \right|/\bar{r}_{i.} \right]}$

In the formulae, $r_{ij}^{*}$ is rank of *i_th_* genotype in the *j_th_* environment based on ($X_{ij}^{*}=X_{ij}-\bar{X}_{i.}$),$\bar{r}_{i.}$ and *M_di_* are the mean and median ranks respectively of the *i*th genotype in the *j*th environment, while $r_{i}^{*}$and $M_{di}^{*}$are obtained from the corrected *X_ij_*.

**Rank-sum**

Kang’s rank-sum (RSM) Yield stability index suggested 3 selection criteria; rank sum (RSM), modified rank sum (MRSM), and statistical yield stability (YSi) (YSi1 and YSi2 are two rank sum statistics that result from sum rank of both yield and Si1 and Si2). In this method, both yield and Shukla’s [15] stability variance are used as selection criteria.

**Ketata’s ranking method**

Ketata et al. [20] proposed rank (kr) method, plotting mean rank across environments against standard deviation (δ_r_) for all genotypes, or plotting mean grain yield (g_y_), across environments against standard deviation (σ_gy_) of ranks for all genotypes

A genotype is considered stable if its kr or gy value is relatively consistent in all the environments. i.e., showing low kr or high gy and having a low σ_r_ [21]. The σr, calculated from the yield rank of genotypes in each environment (r_ij_) based on the uncorrected mean yield values (X_ij_), is expressed as

$$\delta_{r}=\frac{\sqrt{\sum_{j=1}^{n} {(r_{ij}- \overline{r}_{i.})}^{2}}}{n-1}$$

The σgy, calculated from the grain yield of genotypes in each environment (gy_ij_) based on the uncorrected mean yield values (X_ij_), is expressed as

$\sigma_{gy}=\frac{\sqrt{\sum_{j=1}^{n} {(r_{ij}- \overline{X}_{i.})}^{2}}}{n-1}$

**Fox’s TOP-rank stability parameter**

This parameter consists of scoring the number of environments in which each genotype ranked in the top, middle, and bottom third of the trial entries [22]. The genotype with the highest top value is considered a widely adapted genotype.

Fox’s (1990) TOP-rank (TOP) measures of stability for each genotype [22] suggested a superiority measure for general adaptability using the stratified ranking of the cultivars, and ranking was done at each environment separately; the proportion of sites at which the cultivar occurred in the top, middle, and bottom third of the ranks was computed to form the nonparametric measures TOP, MID, and LOW, respectively. A genotype that occurred mostly in the top third (high value of TOP) was considered a widely adapted genotype.

Reference

1. Yan WaKMS: **GGE biplot analysis : a graphical tool for breeders, geneticists, and agronomists**: CRC Press; 2002.

2. Kang MS: **Simultaneous Selection for Yield and Stability in Crop Performance Trials - Consequences for Growers**. *Agronomy Journal* 1993, **85**(3):754-757.

3. Purchase JL, Hatting H, van Deventer CS: **Genotype × environment interaction of winter wheat (*Triticum aestivum L*.) in South Africa: II. Stability analysis of yield performance**. *South African Journal of Plant and Soil* 2000, **17**(3):101-107.

4. Gollob HF: **A statistical model which combines features of factor analytic and analysis of variance techniques**. *Psychometrika* 1968, **33**(1):73-115.

5. GenStat: **GenStat for Windows 14th Edition. VSN International, Hemel Hempstead, UK. Web page: GenStat.co.uk**. In*.*; 2011.

6. Finlay KW, Wilkinson GN: **The analysis of adaptation in a plant-breeding programme**. *Australian Journal of Agricultural Research* 1963, **14**(6):742.

7. Eberhart SA, Russell WA: **Stability Parameters for Comparing Varieties**. *Crop Science* 1966, **6**(1):36-40.

8. Pinthus MJ: **Estimate of genotypic value: A proposed method**. *Euphytica* 1973, **22**(1):121-123.

9. Perkins JM, Jinks JL: **Environmental and genotype-environmental components of variability. 3. Multiple lines and crosses**. *Heredity (Edinb)* 1968, **23**(3):339-356.

10. Bucio Alanis L, Hill J: **Environmental and genotype-environmental components of variability II. Heterozygotes**. *Heredity* 1966, **21**(3):399-405.

11. Francis TR, Kannenberg LW: **Yield Stability Studies in Short-Season Maize. I. A Descriptive Method for Grouping Genotypes**. *Canadian Journal of Plant Science* 1978, **58**(4):1029-1034.

12. Wricke G: **Uber eine Methode zur Erfassung der okologischen Streubreite in Feldverzuchen**. *Z pflanzenzuchtg* 1962, **47**:92-96.

13. Roemer J: **Sinde die ertagdreichen Sorten ertagissicherer**. *Mitt DLG* 1917, **32**(1):87-89.

14. Becker HC, Leon J: **Stability Analysis in Plant-Breeding**. *Plant Breeding* 1988, **101**(1):1-23.

15. Shukla GK: **Some statistical aspects of partitioning genotype-environmental components of variability**. *Heredity (Edinb)* 1972, **29**(2):237-245.

16. Tai GCC: **Genotypic Stability Analysis and Its Application to Potato Regional Trials**. *Crop Science* 1971, **11**(2):184-190.

17. Hanson WD: **Genotypic stability**. *Theoretical and Applied Genetics* 1970, **40**(5):226-231.

18. Nassar R, Huhn M: **Studies on Estimation of Phenotypic Stability - Tests of Significance for Nonparametric Measures of Phenotypic Stability**. *Biometrics* 1987, **43**(1):45-53.

19. Tiiennarasu K: **On Certain Non-Parametric Procedures For Studying Genotype - Environmentinteractions. And Yield Stability**. In*: 1995*.

20. Ketata H, S. Yan, and N. Nachit. : **Relative consistency performance across environments**. In: *International Symposium on Physiology and Breeding of Winter Cereals for stressed Mediterranean Environments: 1989; Montpellier*.

21. Flores F, Moreno MT, Cubero JI: **A comparison of univariate and multivariate methods to analyze G×E interaction**. *Field Crops Research* 1998, **56**(3):271-286.

22. Fox PN, Skovmand B, Thompson BK, Braun HJ, Cormier R: **Yield and Adaptation of Hexaploid Spring Triticale**. *Euphytica* 1990, **47**(1):57-64.
