# Supplementary material for: Integrating univariate and multivariate stability indices for breeding clime-resilient barley cultivars
Source: BMC Plant Biol. 2025 Jan 18;25:76. doi: 10.1186/s12870-024-05530-6 (PMC11748582; doi:10.1186/s12870-024-05530-6)
Supplement: Supplementary file 3 — Supplementary Material 3 [file 12870_2024_5530_MOESM3_ESM.docx]

**Supplementary file 3.** Analysis of variance and Eberhart and Russell’s (1966) analysis for grain yield of 32 barley genotypes grown in 10 test environments.

| **SOV** | **df** | **SS** | **MS** | **F Cal** | **F tab. 1** | **Sig 1%** |
| --- | --- | --- | --- | --- | --- | --- |
| G | 31 | 55.18 | 1.78 | 19294.67 | 1.72 | ** |
| E | 9 | 2142.40 | 238.04 | 2580254.19 | 2.44 | ** |
| G x E | 279 | 291.92 | 1.05 | 11341.35 | 1.26 | ** |
| E + [G x E] | 288 | 2434.32 | 8.45 | 91619.87 | 1.26 | ** |
| Total | 319 | 2489.50 |  |  |  |  |
|  |  |  |  |  |  |  |
| a) E (Linear) | 1 | 2142.40 | 2142.40 | 23222287.74 | 6.68 | ** |
| b) G x E (Linear) | 31 | 53.26 | 1.72 | 18624.50 | 1.72 | ** |
| c) Pooled Deviations | 256 | 238.66 | 0.93 | 10104.98 | 1.27 | ** |
| Genotypes |  |  |  |  |  |  |
| 1 | 8 | 1.89 | 0.24 | 2557.47 | 2.54 | ** |
| 2 | 8 | 8.69 | 1.09 | 11773.26 | 2.54 | ** |
| 3 | 8 | 11.38 | 1.42 | 15413.47 | 2.54 | ** |
| 4 | 8 | 6.51 | 0.81 | 8827.29 | 2.54 | ** |
| 5 | 8 | 5.36 | 0.67 | 7266.61 | 2.54 | ** |
| 6 | 8 | 17.70 | 2.21 | 23986.10 | 2.54 | ** |
| 7 | 8 | 6.54 | 0.82 | 8867.02 | 2.54 | ** |
| 8 | 8 | 7.75 | 0.97 | 10496.22 | 2.54 | ** |
| 9 | 8 | 3.74 | 0.47 | 5064.64 | 2.54 | ** |
| 10 | 8 | 13.08 | 1.63 | 17717.51 | 2.54 | ** |
| 11 | 8 | 4.90 | 0.61 | 6639.75 | 2.54 | ** |
| 12 | 8 | 8.99 | 1.12 | 12179.55 | 2.54 | ** |
| 13 | 8 | 3.20 | 0.40 | 4340.62 | 2.54 | ** |
| 14 | 8 | 10.72 | 1.34 | 14524.25 | 2.54 | ** |
| 15 | 8 | 8.24 | 1.03 | 11164.75 | 2.54 | ** |
| 16 | 8 | 26.83 | 3.35 | 36357.64 | 2.54 | ** |
| 17 | 8 | 6.92 | 0.87 | 9377.73 | 2.54 | ** |
| 18 | 8 | 2.77 | 0.35 | 3747.72 | 2.54 | ** |
| 19 | 8 | 12.25 | 1.53 | 16593.28 | 2.54 | ** |
| 20 | 8 | 5.90 | 0.74 | 7990.75 | 2.54 | ** |
| 21 | 8 | 3.15 | 0.39 | 4269.94 | 2.54 | ** |
| 22 | 8 | 3.39 | 0.42 | 4598.28 | 2.54 | ** |
| 23 | 8 | 4.10 | 0.51 | 5558.68 | 2.54 | ** |
| 24 | 8 | 4.64 | 0.58 | 6291.46 | 2.54 | ** |
| 25 | 8 | 18.25 | 2.28 | 24724.83 | 2.54 | ** |
| 26 | 8 | 10.60 | 1.33 | 14367.68 | 2.54 | ** |
| 27 | 8 | 2.76 | 0.34 | 3735.08 | 2.54 | ** |
| 28 | 8 | 5.24 | 0.66 | 7106.01 | 2.54 | ** |
| 29 | 8 | 4.04 | 0.51 | 5479.79 | 2.54 | ** |
| 30 | 8 | 4.24 | 0.53 | 5751.28 | 2.54 | ** |
| 31 | 8 | 3.52 | 0.44 | 4769.71 | 2.54 | ** |
| 32 | 8 | 1.34 | 0.17 | 1821.12 | 2.54 | ** |
| Poled error | 620 | 0.057 | 9.2256E-05 |  |  |  |
