# Supplementary material for: Integrating univariate and multivariate stability indices for breeding clime-resilient barley cultivars
Source: BMC Plant Biol. 2025 Jan 18;25:76. doi: 10.1186/s12870-024-05530-6 (PMC11748582; doi:10.1186/s12870-024-05530-6)
Supplement: Supplementary file 4 — Supplementary Material 4 [file 12870_2024_5530_MOESM4_ESM.docx]

**Supplementary file 4.** Spearman's rank correlation coefficients among stability parameters and grain yield for 32 barley genotypes tested in ten environments.

|  | *GY* | *CV%* | *b_i_* | *S^2^_di_* | *R_2_* | *σi^2^* | *B_i_* | *D_ji_* | *W^2^_i_* | *Sxi^2^* | *α_i_* | *λ_i_* | *D_i_^2^* | *ASV* | *SI ^3^* | *SI ^6^* | *NP I ^(3)^* | *NP I ^(4)^* | *RSM* | *YSI* | *δ_r_* | *k_r_* | *TOP* |
| --- | --- | --- | --- | --- | --- | --- | --- | --- | --- | --- | --- | --- | --- | --- | --- | --- | --- | --- | --- | --- | --- | --- | --- |
| GY | 1 |  |  |  |  |  |  |  |  |  |  |  |  |  |  |  |  |  |  |  |  |  |  |
| CV% | 0.03 | 1 |  |  |  |  |  |  |  |  |  |  |  |  |  |  |  |  |  |  |  |  |  |
| b_i_ | 0.66 | 0.70 | 1 |  |  |  |  |  |  |  |  |  |  |  |  |  |  |  |  |  |  |  |  |
| S^2^_di_ | 0.00 | 0.34 | 0.03 | 1 |  |  |  |  |  |  |  |  |  |  |  |  |  |  |  |  |  |  |  |
| R^2^ | 0.27 | 0.03 | 0.42 | -0.86 | 1 |  |  |  |  |  |  |  |  |  |  |  |  |  |  |  |  |  |  |
| σ_i_^2^ | 0.09 | 0.28 | 0.05 | 0.93 | -0.84 | 1 |  |  |  |  |  |  |  |  |  |  |  |  |  |  |  |  |  |
| B_i_ | 0.66 | 0.70 | 1.00 | 0.03 | 0.42 | 0.05 | 1 |  |  |  |  |  |  |  |  |  |  |  |  |  |  |  |  |
| D_ji_ | 0.00 | 0.34 | 0.03 | 1.00 | -0.86 | 0.93 | 0.03 | 1 |  |  |  |  |  |  |  |  |  |  |  |  |  |  |  |
| W^2^_i_ | 0.09 | 0.28 | 0.05 | 0.93 | -0.84 | 1.00 | 0.05 | 0.93 | 1 |  |  |  |  |  |  |  |  |  |  |  |  |  |  |
| S_xi_^2^ | 0.65 | 0.74 | 0.96 | 0.27 | 0.18 | 0.32 | 0.96 | 0.27 | 0.32 | 1 |  |  |  |  |  |  |  |  |  |  |  |  |  |
| α_i_ | 0.66 | 0.70 | 1.00 | 0.03 | 0.42 | 0.05 | 1.00 | 0.03 | 0.05 | 0.96 | 1 |  |  |  |  |  |  |  |  |  |  |  |  |
| λ_i_ | 0.00 | 0.34 | 0.03 | 1.00 | -0.86 | 0.93 | 0.03 | 1.00 | 0.93 | 0.27 | 0.03 | 1 |  |  |  |  |  |  |  |  |  |  |  |
| D_i_^2^ | 0.00 | 0.34 | 0.03 | 1.00 | -0.86 | 0.93 | 0.03 | 1.00 | 0.93 | 0.27 | 0.03 | 1.00 | 1 |  |  |  |  |  |  |  |  |  |  |
| ASV | -0.13 | -0.20 | -0.16 | -0.29 | 0.14 | -0.34 | -0.16 | -0.29 | -0.34 | -0.24 | -0.16 | -0.29 | -0.29 | 1 |  |  |  |  |  |  |  |  |  |
| SI ^3^ | 0.49 | -0.12 | 0.19 | 0.43 | -0.34 | 0.51 | 0.19 | 0.43 | 0.51 | 0.32 | 0.19 | 0.43 | 0.43 | -0.12 | 1 |  |  |  |  |  |  |  |  |
| SI ^6^ | 0.61 | -0.19 | 0.25 | 0.29 | -0.20 | 0.38 | 0.25 | 0.29 | 0.38 | 0.33 | 0.25 | 0.29 | 0.29 | -0.09 | 0.96 | 1 |  |  |  |  |  |  |  |
| NP I ^(3)^ | 0.72 | -0.07 | 0.37 | 0.29 | -0.18 | 0.45 | 0.37 | 0.29 | 0.45 | 0.47 | 0.37 | 0.29 | 0.29 | -0.21 | 0.87 | 0.90 | 1 |  |  |  |  |  |  |
| NP I ^(4)^ | 0.82 | -0.38 | 0.28 | -0.12 | 0.18 | -0.05 | 0.28 | -0.12 | -0.05 | 0.26 | 0.28 | -0.12 | -0.12 | -0.10 | 0.57 | 0.72 | 0.75 | 1 |  |  |  |  |  |
| RSM | -0.62 | 0.15 | -0.48 | 0.57 | -0.77 | 0.61 | -0.48 | 0.57 | 0.61 | -0.30 | -0.48 | 0.57 | 0.58 | -0.11 | -0.01 | -0.19 | -0.15 | -0.61 | 1 |  |  |  |  |
| YSI | 0.80 | -0.10 | 0.45 | 0.19 | 0.01 | 0.27 | 0.45 | 0.19 | 0.27 | 0.50 | 0.45 | 0.19 | 0.19 | -0.18 | 0.66 | 0.75 | 0.79 | 0.78 | -0.40 | 1 |  |  |  |
| δ_r_ | 0.07 | 0.01 | -0.03 | 0.59 | -0.59 | 0.65 | -0.03 | 0.59 | 0.65 | 0.15 | -0.03 | 0.59 | 0.60 | -0.04 | 0.86 | 0.73 | 0.61 | 0.11 | 0.40 | 0.31 | 1 |  |  |
| k_r_ | -0.87 | 0.30 | -0.38 | 0.07 | -0.18 | -0.01 | -0.38 | 0.07 | -0.01 | -0.36 | -0.38 | 0.07 | 0.07 | 0.10 | -0.59 | -0.74 | -0.78 | -0.98 | 0.61 | -0.78 | -0.15 | 1 |  |
| TOP | 0.69 | -0.29 | 0.28 | 0.00 | 0.09 | 0.06 | 0.28 | 0.00 | 0.06 | 0.29 | 0.28 | 0.00 | 0.00 | 0.02 | 0.75 | 0.88 | 0.80 | 0.86 | -0.48 | 0.74 | 0.42 | -0.88 | 1 |

Gen.; Genotype, GY; Grain yield (tons ha^−1^), CV; Coefficient of variability, b_i_; Linear regression coefficients, S^2^_di_; Deviations from regression, R^2^; Coefficient of determination, σ**_i_^2^**; Stability variance, *β_i_*; Perkins and Jinks’s stability parameters, W^2^_i_; Wricke ecovalence, S_xi_^2^; Roemer’s environmental variance, λ_i_ and *α_i_*; Tai’s stability statistics, D_i_^2^; Hanson genotypic stability, ASV; AMMI stability value, RSM; Kang’s rank-sum, YSI; Yield stability index, δ r, and kr; Ketata’s plotting mean rank, TOP; Fox’s TOP-rank stability parameter.
